# A 16-week progressive exercise training intervention in treatment-naïve chronic lymphocytic leukaemia: a randomised-controlled pilot study

**DOI:** 10.3389/fonc.2024.1472551

**Published:** 2024-12-05

**Authors:** Frankie F. Brown, Rebecca Oliver, Rachel Eddy, Adam J. Causer, Annabelle Emery, Harrison D. Collier-Bain, David Dutton, Josephine Crowe, Daniel Augustine, John Graby, Daniel Rees, Daniela Rothschild-Rodriguez, Oliver J. Peacock, Sally Moore, James Murray, James E. Turner, John P. Campbell

**Affiliations:** 1 Department for Health, University of Bath, Bath, United Kingdom; 2 School of Applied Sciences, Edinburgh Napier University, Edinburgh, United Kingdom; 3 Department for Haematology, Royal United Hospitals Bath NHS Foundation Trust, Bath, United Kingdom; 4 Department for Haematology, Great Western Hospitals NHS Foundation Trust, Swindon, United Kingdom; 5 Department for Cardiology, Royal United Hospitals Bath NHS Foundation Trust, Bath, United Kingdom; 6 School of Biological Sciences, University of Southampton, Southampton, United Kingdom; 7 School of Sport, Exercise and Rehabilitation Sciences, University of Birmingham, Birmingham, United Kingdom; 8 School of Medical and Health Science, Edith Cowan University, Perth, WA, Australia

**Keywords:** CLL (chronic lymphocytic leukaemia), exercise intervention, T cells, lean mass, CD49d expression, CD38

## Abstract

**Background:**

Chronic lymphocytic leukaemia (CLL) typically presents with asymptomatic, early-stage disease that is monitored until disease progression (‘treatment-naïve’ CLL). The objective of this pilot study was to assess the feasibility and preliminary safety of an exercise program in treatment-naïve CLL. We also sought to preliminarily assess the impact of the exercise program on disease activity, as it has been proposed that exercise training may reduce disease outgrowth in treatment-naïve CLL.

**Methods:**

A total of 40 treatment-naïve CLL patients were recruited into this randomised-controlled pilot study, and after screening, n = 28 were randomised into a 16-week, home-based, partially supervised, personalised, progressive exercise intervention (*n* = 14: mean ± SD: age = 62 ± 12 years) or 16 weeks of usual care, control group (*n* = 14: mean ± SD: age = 61 ± 10 years). The primary outcome measures were safety (number and severity of adverse events) and feasibility (uptake, retention, and adherence to the trial). Disease activity (CD5^+^/CD19^+^ CLL cells clonally restricted to kappa or lambda) and other immune cell phenotypes, with a principal focus on T cells, were measured by flow cytometry. Other secondary outcomes included DEXA-derived body composition, cardiorespiratory and functional fitness, resting cardiovascular measures.

**Results:**

Trial uptake was 40%, and the overall retention rate was 86%, with 79% of the exercise group and 93% of the control group completing the trial. Adherence to the exercise intervention was 92 ± 8%. One serious adverse event was reported unrelated to the trial, and one adverse event related to the trial was reported. The exercise intervention elicited a 2% increase in DEXA-derived lean mass in the exercise group compared with a 0.4% decrease in the control group (*p* = 0.01). No between-group differences were observed over time for whole-body mass, BMI, bone mineral density, body fat, blood pressure resting heart rate, or measures of cardiorespiratory or functional fitness (all *p* > 0.05). No between-group differences were observed over time for clonal CLL cells and CD4^+^ or CD8^+^ T-cell subsets (all *p* > 0.05).

**Conclusion:**

The exercise training program used in this study was feasible in people with treatment-naïve CLL who passed pre-trial screening, and we preliminarily conclude that the exercise training program was safe and also resulted in an increase in lean mass.

**Clinical trial registration:**

**https://doi.org/10.1186/ISRCTN55166064, identifier ISRCTN 55166064.**

## Introduction

Chronic lymphocytic leukaemia (CLL) is the most common adult leukaemia in the UK, and it has an estimated annual incidence of 7.6 per 100,000 people (1). CLL is most common among people aged ≥60 years (1). Most newly diagnosed CLL patients present with asymptomatic, early-stage disease (2, 3). Clinical trials investigating early therapeutic intervention have not reported improved clinical outcomes in patients with early-stage, asymptomatic CLL when compared with the current gold standard approach of clinical observation without therapy, referred to as ‘watch and wait’, or active surveillance. Thus, ‘treatment-naïve’ patients are monitored without anti-CLL treatment, until there is evidence of progressive or symptomatic disease, referred to as ‘active disease’ (3–6).

There is growing evidence from preclinical and human epidemiology studies of multiple cancer types that physical activity can delay or avert the outgrowth of cancers (7–9), in a mechanistic process that may involve exercise-induced alterations to anticancer immunity (10). This has stimulated numerous studies to explore the effects of exercise training on early-stage cancer outgrowth (11–17). In the context of CLL, it has recently been proposed that short-term exercise training might suppress disease outgrowth in treatment-naïve CLL (12). Specifically, an exploratory, non-randomised, pilot study (*n* = 10 exercise group *versus n* = 6 control group) of supervised (in-person) exercise training combining high-intensity interval (walking) training (HIIT) and resistance training for 12 weeks reported that the absolute change in blood CD5^+^CD19^+^ B-CLL cells over time was 21.4% less in the exercise group compared with the control group. The aforementioned study also concluded that the exercise training used was safe and feasible, and compliance to the exercise intervention was associated with physiological adaptations including to muscle strength and immune function (12). In a separate study, Crane et al. (13) investigated a 16-week, home-based, unsupervised aerobic and/or resistance exercise intervention aimed at increasing leisure-time physical activity in *n* = 24 CLL patients, of which 50% were receiving targeted therapy. Crane et al. (13) reported that the intervention increased leisure-time physical activity and reduced fatigue among exercisers. Additionally, it was reported that the reduction in fatigue at 16 weeks correlated with changes to immune phenotypes in blood (increased CD4:CD8 ratio and lower percentage of HLA^-^DR^+^PD-1^+^CD4^+^ T-cells); however, no B-CLL cell counts were reported (13). As reported by Lee et al. (18), the intervention was deemed feasible with program completion rates of 90% for aerobic exercise alone and 76.5% for resistance and aerobic exercise (18). It has been shown in other haematological cancers that 12 weeks of moderate-intensity aerobic training improved physical function, fatigue, cardiorespiratory fitness, and quality of life (19), whereas 36 weeks of exercise training was effective at improving balance and quality of life (20). Nevertheless, given the paucity of randomised-controlled studies investigating the effects of exercise training in treatment-naïve CLL, further research is warranted to assess the potential impact of exercise training on disease activity and physiological and biological outcomes, including immune function. Before doing so in a larger trial, it is important that the safety and feasibility of exercise training in people with treatment-naïve CLL is assessed in a randomised-controlled pilot study. We therefore sought to deliver an individually prescribed, progressive, partially supervised, exercise training program in treatment-naïve CLL comprising aerobic exercise that increased from moderate-to-vigorous intensity exercise, which also included resistance, balance, and flexibility exercises in line with exercise recommendations for older adults (21). The primary aim of this study was to investigate the safety and feasibility of an exercise program in people with treatment-naïve CLL. We also sought to explore the effects of the exercise training program on blood CLL cell counts and other immunophenotypes, physical function, body composition, and wellbeing outcomes in treatment-naïve CLL.

## Materials and methods

### Study design

This randomised-controlled pilot study included measurements made in week 0 (pre intervention) and week 17 (post intervention). Following the measurements in week 0, participants were randomised into an exercise group or a control group. Participants randomised into the exercise group were prescribed a 16-week home-based partially supervised progressive exercise program. Participants randomised into the control group received usual clinical care throughout the 16 weeks. The two measurement visits took place at the University of Bath. The protocol was approved by the London - Dulwich NHS Research Ethics Committee (reference 292564) and the University of Bath Research Ethics Approval Committee for Health (reference EP 20/21 107). The study was prospectively registered (ISRCTN 55166064).

### Participants

**Figure 1:**
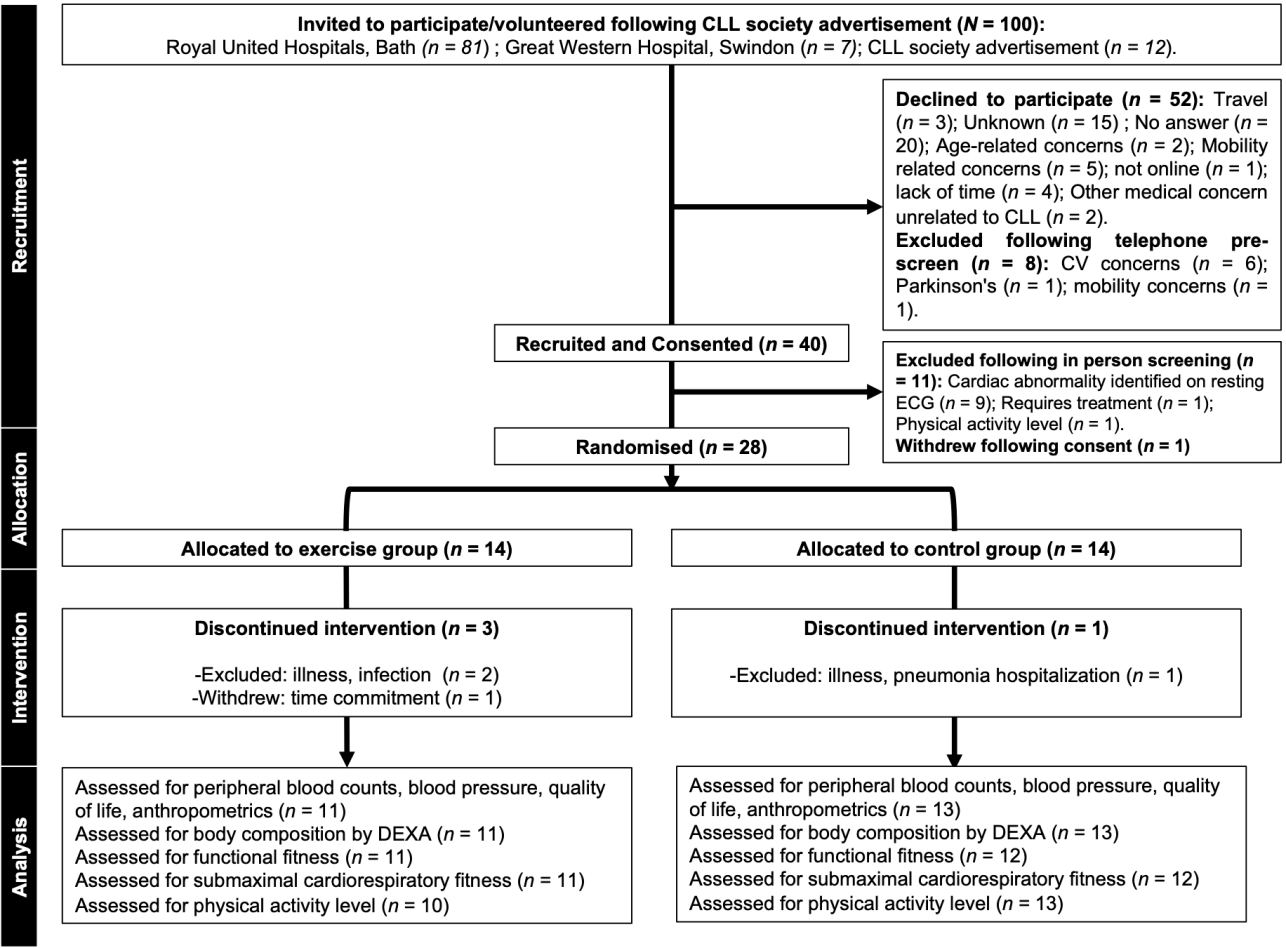
CONSORT diagram showing flow of participants through the pilot trial. Missing data: Physical activity level (n=1 participant declined post visit physical activity monitoring); functional fitness (n=1 unable to perform upper body flexibility due to shoulder pain); submaximal cardiorespiratory fitness (n=1 participant did not complete the submaximal cardiorespiratory fitness test at the post visit due to discomfort). CLL, chronic lymphocytic leukaemia; CV, cardiovascular; ECG, electrocardiography; DEXA, dual-energy X-ray absorptiometry.

Recruitment was open for 15 months between October 2021 and December 2022. During this 15-month period, 100 patients with asymptomatic, early-stage, treatment-naïve CLL and aged ≥18 years were invited to participate. Diagnosis of CLL was in line with the International Workshop on CLL (iwCLL) guidelines, based on the presence of ≥5 × 10^9^ B cells/L blood, sustained for ≥3 months, and confirmed by blood smear, immunophenotyping, and/or cytogenetics (3). Of the *n* = 100 treatment-naïve CLL patients approached, *n* = 52 declined to participate in the trial (**Figure 1**). Initial telephone screening involving verbal completion of a physical activity readiness questionnaire (PAR-Q) resulted in *n* = 8 patients being excluded (**Figure 1**). Therefore, *n* = 40 (40% uptake) treatment-naïve CLL patients attended an in-person screening visit (**Figure 1**). During the screening visit, patients were assessed for the following exclusion criteria: severe/uncontrolled cardiovascular, neurological, psychiatric, metabolic, and respiratory disease, musculoskeletal conditions that would impair the ability to cycle, metastatic/palliative malignancy, abnormal haematological results (e.g., haemoglobin <100 g/dL) and ECOG performance status >1. Physically active patients were excluded, and this was assessed by verbal confirmation using the International Physical Activity Questionnaire (IPAQ); ‘physically active’ is defined by the World Health Organisation as ‘a physical activity level to meet present physical activity recommendations’. Current physical activity guidelines for adults are at least 150 min–300 min of moderate-intensity aerobic physical activity, or at least 75 min–150 min of vigorous-intensity aerobic physical activity, or an equivalent combination of both throughout the week (22). A 12-lead resting electrocardiogram (ECG) was recorded and reviewed by a cardiologist. All *n* = 40 patients provided written informed consent during the screening visit and were subsequently recruited into the trial.

### Measurement visit

#### Resting measurements and body composition

Participants attended the measurement visit between 06:45 and 11:00 having not undertaken strenuous exercise and having not consumed alcohol for 24 h, caffeine for ≥8 h, and having fasted for ≥8 h. Height was assessed by a stadiometer (Seca; Birmingham, United Kingdom), and body mass was determined by electronic scales (Tanita InnerScan; Tokyo, Japan). Body composition was estimated via whole-body dual-energy x-ray absorptiometry (DEXA) scan. Participants consumed 500 mL of water before arriving at the laboratory and voided their bladder prior to the scan. Next, participants were positioned supine (Discovery, Hologic; Bedford, UK) with feet equally spaced and arms with an even gap from the trunk. Whole-body composition analysis was performed with regions sectioned as recommended by the manufacturer (Hologic; Bedford, UK). Participants rested in a supine position for ~30 min prior to three blood pressure measurements using an automated blood pressure monitor (SunTech Tango M2; Wuxi, China) and a 50 mL blood sample being collected from an antecubital vein by venepuncture.

#### Functional fitness and wellbeing measures

Dynamic balance was measured as time to complete the 8 Foot Up and Go Test. Lower body muscle strength was measured as the number of repetitions completed in a 30-s sit-to-stand test. Upper-limb flexibility was measured using the back scratch test, and lower-limb flexibility was measured using the sit-and-reach test. Upper-body strength was measured using a handgrip dynamometer (Takei 5401 Grip D; Niigata City, Japan). Wellbeing was measured using a battery of validated questionnaires. Health-related quality of life was assessed via the 30-item European Organisation for Research and Treatment of Cancer Quality of Life Questionnaire C30 (EORTC QLQ-C30) and the 16-item CLL-specific module (EORTC QLQ-CLL16). Sleep quality was quantified via Pittsburgh Sleep Quality Index (PQSI) and fatigue via Functional Assessment of Chronic Illness Therapy (FACIT)-Fatigue scale. Perceived stress was assessed via the Perceived Stress Scale (PSS). Satisfaction with life was measured via the Satisfaction With Life Scale.

#### Cardiopulmonary exercise test (anaerobic threshold test)

Anaerobic threshold (AT) was determined by a submaximal ramp incremental cardiopulmonary exercise test (CPET) on a cycle ergometer (Lode Excalibur; Groningen, The Netherlands). Participants were instructed to maintain a cadence between 60 rpm and 80 rpm throughout and completed a 3-min warm-up of unloaded cycling, followed by a 5 W·min^−1^–25 W·min^−1^ incremental phase up to a rating of perceived exertion (RPE) of ≥17 on a Borg 6-20 Scale (26), or until the researchers were satisfied that AT was achieved (e.g., RER >1.0), and then an unloaded cooldown for 5 min. Breath-by-breath gas exchange/ventilation (Carefusion Vyntus CPX; CA, United States), heart rate (HR) via 12-lead electrocardiogram (Carefusion Vyntus ECG; CA, United States), and arterial oxygen saturation (SpO_2_) via pulse oximetry (Nonin PureSAT; MN, United States) were recorded continuously during exercise, whereas RPE was recorded every 60 s and blood pressure was recorded pre- and post-incremental exercise. Pulmonary oxygen uptake (VO_2_), carbon dioxide production (VCO_2_), and ventilatory equivalents of O_2_ (V_E_/VO_2_) and CO_2_ (V_E_/VCO_2_) data were interpolated to 15-s averages. The V-slope method (23) was used to determine AT, independently by two researchers, and was further confirmed through visual inspection of V_E_/VO_2_ and V_E_/VCO_2_. AT was reported in terms of VO_2_ (mL·kg^−1^·min^−1^), in terms of power output (W), and as a percentage of age-predicted maximum heart rate (HR_max_), using the following equation:


$$\%\;HR_{max}=\;(measured\;heart\;rate\;÷\;(220-age\;in\;years))\;\times \;100$$

#### Blood sample processing

Blood was collected into sodium heparin- (17 IU/mL), ethylenediaminetetraacetic acid- (EDTA, 1.8 mg/mL), and silica act clot activator-treated vacutainers (Becton Dickinson, BD; NJ, United States). Peripheral blood mononuclear cells (PBMCs) were isolated from sodium heparin-treated blood—diluted 1:1 with phosphate-buffered saline (PBS; KCl 0.2 g/L, KH_2_PO_4_ 0.2 g/L, NaCl 8.0 g/L, Na_2_HPO_4_ 1.15 g/L; without CaCl_2_ and MgCl_2_ herein) (Sigma-Aldrich; MI, United States) containing 2% (v/v) heat-inactivated foetal calf serum (HI-FCS) (Gibco; MA, United States)—in SepMate™ tubes (StemCell Technologies; Vancouver, Canada) that were pre-loaded with 15-mL Ficoll-Paque™ PLUS Media (Cytiva; MA, United States). SepMate™ tubes were centrifuged at 1,200 × g for 10 min at room temperature, and the isolated cells were transferred to a 50-mL conical tube (Falcon; GA, United States). PBMCs were washed twice by centrifugation at 500 × g for 5 min at room temperature and resuspended in 20 mL–50 mL PBS (2% HI-FCS), depending on the density of the cell suspension. PBMCs were used for immunophenotyping lymphocyte subsets by flow cytometry.

### Blood immunophenotyping

#### Full blood counts

EDTA-treated whole blood was analysed in triplicate using an automated haematology analyser (Sysmex KX-21N; Kobe, Japan) for leukocyte and erythrocyte numbers, haemoglobin, haematocrit, platelets, and proportions/numbers of lymphocytes, monocytes, neutrophils, eosinophils, basophils, and immature granulocytes. In *n* = 14 participants, lymphocyte and monocyte proportions/number could not be determined as B-CLL cells presented in a monocyte-like morphology. To compute a value for MNCs, a flow cytometric approach was used in combination with other leukocyte differential data. Using flow cytometry in an FSC-A × SSC-A gate, a large MNC gate was drawn around the lymphocytes and monocytes, and those MNC proportions/numbers were calculated by negative deduction thereafter using the following equation utilising whole blood count data acquired by the Sysmex analyser:


Mononuclear cell (MNC) count (Monocytes and Lymphocytes)= Total White Blood Cell Count −neutrophils− eosinophils−basophils−immature granulocytes

#### Immunophenotyping leukocyte subsets

Peripheral blood mononuclear cells (PBMCs) previously isolated from sodium heparin-treated blood were resuspended in MACS buffer (PBS, 10% [v/v] HI-FCS, and 2 mM EDTA [Invitrogen]) at a concentration of 1 × 10^7^ cells/mL, and 1 × 10^6^ cells were seeded in 5-mL round-bottom polystyrene test tubes (Falcon; GA, United States). Viability was determined in the B-CLL tube using a fixable viability stain 575 (FVS575 PE-CF594). PMBCs were stained in brilliant stain buffer (Becton Dickinson, BD; NJ, United States) for 30 min in the dark at room temperature with antibody-fluorochrome cocktails of two panels to investigate either B-CLL (anti–CD3 PE-Cy5.5 [SK7], anti–CD5 PE-Cy7 [L17F12], anti–CD19 R718 [SJ2SC1], anti–kappa FITC [G20-193], anti–lambda PE [JDC-12], anti–CD38 BV421 [HIT2], anti–CD45 V500 [HI30], anti–CD49d APC [9F10], anti–sIgM BV605 [G20-127], and anti–CXCR4 BB700 [12G5]) or T-cell (anti–CD3 BV510 [SK7], anti–CD4 PE-Cy7 [SK3], anti–CD8 APC-H7 [SK1], anti–CD27 BV605 [L128], anti–CD45RA BB515 [HI100], anti–PD1 BB700 [EH12.1], anti–TIM3 BV650 [7D3], anti–CD25 R718 [2A3], anti–CD127 PE [hIL-7R-M21], anti–CD95 BV421 [DX2], anti–CTLA-4 PE-CF594 [BNI3], and anti–CD19 BUV496 [SJ26C1] subsets. Labelled cells were then centrifuged at 500 × g for 5 min at room temperature. The B-CLL tube was then fixed with 2% PFA for 10 min at 4°C, centrifuged at 500 × g for 5 min at room temperature, and then resuspended in 500 µL MACS buffer and stored at 4°C until analysis. The T-cell tube was fixed and permeabilised (Invitrogen, Thermo Fisher Scientific; MA, United States) for 60 min at 4°C; following the permeabilisation, the T-cell PBMCs were resuspended in 2 mL buffer (Invitrogen, Thermo Fisher Scientific; MA, United States), centrifuged at 500 × g for 5 min at room temperature, and then labelled with anti-FoxP3 AF647 [259D/C7] for 30 min in the dark at room temperature. Following the internal staining, the T-cell PBMCs were resuspended with 2 mL of buffer and centrifuged at 500 × g for 5 min at room temperature twice, and then resuspended in 500 µL MACS buffer and stored at 4°C until analysis. Samples were analysed using a Fortessa X-20 flow cytometer and FACSDiva software (Becton Dickinson, BD; NJ, United States) within 24 h of preparation.

All antibodies were purchased from BD Biosciences (Becton Dickinson, BD; NJ, United States) with the exception of anti-CD3 PE-Cy5.5 [SK7], which was purchased from Invitrogen (Invitrogen, Thermo Fisher Scientific; MA, United States). Antibodies were pre-titrated to ensure that optimal fluorescent staining was achieved (data not shown). Both unstained cells from each participant, and single stained tubes containing anti-mouse positive (Igκ) and negative control compensation particles (Becton Dickinson, BD; NJ, United States) were used in each assay to account for spectral overlap. Data were analysed in FlowJo (Becton Dickinson, BD; NJ, United States) according to the representative gating strategies reported in **Supplementary Figures 1**–**3**.

#### Habitual device measured physical activity

Habitual physical activity was measured for 7 days following each measurement visit. Physical activity measurements were derived from the raw acceleration data measured by triaxial accelerometers (GENEActiv, Activinsights Ltd.; Kimbolton, UK) placed on the non-dominant wrist. A sampling rate of 60 Hz was used. The raw data were processed with the R-package GGIR (version 2.8-0) (24) applying auto-calibration (25). The raw triaxial acceleration signals were transformed to the Euclidean Norm Minus One (ENMO; expressed in milli gravitational units mg), which is an omnidirectional metric used to quantify acceleration of movement (26, 27). Here, the gravitational acceleration (1 g = 9.81 m/s^2^) is subtracted from the vector magnitude of the three axes to correct for gravity. Negative values are rounded up to 0. The ENMO metric was further aggregated into 1-min epochs. Each 1-min epoch was then categorised into the following physical activity intensities based on the ENMO cut-off values (28) proposed by Hildebrand et al. (29, 30): sedentary (< 45.8 mg), light physical activity (LPA) (45.8 mg–93.2 mg), moderate physical activity (MPA) (93.2 mg–418.3 mg), and vigorous physical activity (VPA) (≥ 418.3 mg). Based on this categorisation, the average weekly duration (in minutes) of MVPA (sum of MPA and VPA) was calculated for each participant.

#### Randomisation

Participants were stratified by age (>60, <60) and biological sex (male, female) and underwent randomisation using restricted blocks of random size with an allocation of 1: 1 into the exercise group (*n* = 14) or the control group (*n* = 14). The password-protected web-based platform sealed envelope was used to generate the trial randomisation sequence and for individual participant randomisations (31). Generation and storage of the trial randomisation sequence was by a member of the research team that was not involved in any participant facing activities, ensuring allocation concealment.

### Intervention procedures

#### Exercise program

Participants randomised into the exercise group were prescribed a home-based 16-week personalised exercise program. An upright cycle ergometer (Vision Upright Bike, U60, Johnson Health Tech UK Ltd; Staffordshire, UK), blood pressure monitor (Omron M2, Omron Healthcare; Milton Keynes, UK), and resistance bands (Meglio; Reading, UK) were provided. The program comprised two live online supervised sessions of aerobic and resistance exercise and one home-based aerobic exercise session per week (**Supplementary Figure 4**).

#### Supervised exercise training

Supervised sessions were performed at home and were supervised online, via the Microsoft Teams platform (Microsoft Corporation; WA, United States), by an exercise physiologist. Supervised sessions included a safety pre-screen step of a resting blood pressure reading. Following the safety pre-screen, participants completed a 5-min unloaded warm-up. Supervised aerobic exercise was a 30-min cycle, performed as three 10-min bouts. Each bout involved 8 min of cycling (‘exercise interval’) at workload intensities (Watts (W)) prescribed from the anaerobic threshold (AT) test assessed during the measurement visit, and participants were instructed to maintain a cadence of 60 rpm throughout the 8-min exercise interval, followed by 2 min of unloaded cycling. The exercise interval was prescribed at a moderate intensity initially to allow safety to be evaluated prior to progressing to vigorous intensities. A similar design has been previously shown to be safe and feasible in people with MGUS and smouldering myeloma (11). Specifically, the intensity of the exercise interval progressed from a workload of watts at −5% AT (weeks 1–2), to watts at AT (weeks 3–6), to watts at +5% AT (weeks 7–10), and finally to watts at +15% AT (weeks 11–16) (**Supplementary Figure 4**). Heart rate was continuously monitored (Vision Upright Bike, U60, Johnson Health Tech UK Ltd; Staffordshire, UK), and RPE was recorded using a 6-20 Likert scale (32) at the end of each 8-min exercise interval.

Following aerobic exercise, supervised resistance exercises were completed for six major muscle groups (reverse fly, chest fly, triceps extension, squat, calf raise, and abduction). Resistance was applied with elastic bands (Meglio, Reading, UK). Familiarisation was performed in week 1 by performing two sets of 10 repetitions using elastic bands with increasing resistance to inform the starting resistance level. Progression comprised two sets of 12 repetitions (weeks 3–6), and then three sets of 10 repetitions (weeks 7–10), and finally four sets of 10 repetitions (weeks 11–16). Resistance was increased at each progression point (**Supplementary Figure 4**).

#### Unsupervised exercise training

Home-based exercise consisted of one weekly 45-min walk at a moderate intensity (RPE 12-13/20) (33) where duration was recorded with a wrist-based fitness tracker (Garmin Forerunner 15, Garmin; Southampton, UK) and reported using a home exercise diary and verbally during the next supervised online session. Participants were provided with NHS balance exercises (34) and flexibility exercises to perform daily. Flexibility exercises were static stretches of the pectorals, deltoids, triceps, latissimus dorsi, adductors, gastrocnemius, quadriceps, and hamstrings.

#### Intervention measurements

Adherence to the intervention was reported as the proportion of online supervised exercise sessions attended and home-based exercise sessions completed using exercise record cards completed by the exercise physiologist and diaries/verbal confirmation, respectively. Compliance to supervised aerobic exercise was assessed by the observed completion of each exercise interval by the supervising exercise physiologist. Each intensity progression was defined as feasible if >70% of participants completed >75% of sessions at the prescribed intensity (12). Compliance to supervised resistance exercise was assessed by comparing the average number of repetitions performed per exercise per session, to the target repetition prescription.

Participants were provided with a wrist-based fitness tracker (Garmin Forerunner 15, Garmin; Southampton, UK)) for the duration of the study. Compliance to the duration of home-based walks was determined by comparing the duration reported in exercise diaries/verbally during the online sessions to the prescribed duration. Compliance to the intensity of home-based walks was monitored by comparing the RPE reported in exercise diaries to the prescribed RPE. Safety was reported as the incidence, severity, expectedness, and relatedness of adverse events categorised by haematologists according to Common Terminology Criteria for Adverse Events guidelines (v5.0).

#### End of intervention

Following the 16-week intervention, participants from both the exercise and control groups returned to the laboratory for the post-intervention measurement visit. During this visit, the pre-intervention measurements were repeated.

### Statistical analysis

Statistical analysis was performed using GraphPad Prism (version 8, GraphPad Software, California, USA) and SPSS Statistics (version 27, IBM SPSS Statistics for Windows, New York, USA). Data are presented as mean ± standard deviation (SD) unless otherwise stated. Normality was assessed by Shapiro–Wilk’s test of normality on the studentised residuals. Baseline characteristics, body composition, resting cardiovascular measurements, cardiorespiratory fitness, and functional fitness tests were all normally distributed (all *p* > 0.05). Quality of life and blood phenotype measurements were not normally distributed (all *p* < 0.05). Independent samples T-tests were used to determine differences in baseline characteristics between the exercise and control groups. In addition, two-way repeated measures analysis of variance (ANOVA), which has been shown to be robust to non-normally distributed data (35), was used to identify main effects of time (e.g., pre-intervention vs. post-intervention) and between groups (i.e., Exercise vs. Control), and interaction effects.

## Results

### Baseline characteristics

A total of 100 (*n* = 100) treatment-naïve CLL patients (Binet stages A and B) were approached. Trial uptake was 40%, and *n* = 40 participants with treatment-naïve CLL took part in screening. Following preliminary assessment of participant suitability for exercise, *n* = 11 participants were excluded (the majority of these, *n* = 9, were due to cardiac abnormalities identified during safety screening) and *n =* 1 withdrew. Consequently, *n* = 28 participants were randomised into a 16-week, home-based, partially supervised, personalised, progressive exercise intervention (*n* = 14: mean ± SD: age = 62 ± 12 years) or 16 weeks of usual care, control group (*n* = 14: mean ± SD: age = 61 ± 10 years). The overall retention rate was 86%, with 79% (*n* = 11) of the exercise group and 93% (*n* = 13) of the control group completing the trial. Reasons for withdrawing and exclusion during the trial are shown in **Figure 1**. There were no significant differences between groups for baseline participant characteristics (age, height, body mass, BMI, and physical activity level) (all *p* > 0.05) (**Table 1**).

**Table 1:** Baseline participant characteristics.

	Exercise (*n* =14)		Control (*n* =14)		T-test statistic
Age (years)	62 ± 12		61 ± 10		*t*(26) = 0.24, *p* = 0.81
Height (m)	169.7 ± 7.3		169.8 ± 8.9		*t*(26) = −0.02, *p* = 0.99
Body mass (kg)	74.5 ± 17.3		80.8 ± 18.3		*t*(26) = −0.93, *p* = 0.36
BMI (kg/m^2^)	25.7 ± 4.9		27.8 ± 5.0		*t*(26) = −1.13, *p* = 0.27
Physical activity level (minutes/week)					
Sedentary	700.4 ± 132.1		736.4 ± 87.4		*t*(26) = −0.85, *p* = 0.40
Light	176.5 ± 61.6		195.4 ± 107.9		*t*(26) = −0.57, *p* = 0.58
Moderate	130.6 ± 59		112.1 ± 50		*t*(26) = 0.90, *p* = 0.38
Vigorous	3.1 ± 5.2		0.5 ± 1.2		*t*(26) = 1.86, *p* = 0.08
MVPA	133.7 ± 62		112.6 ± 50		*t*(26) = 0.99, *p* = 0.33
	*N*	Percentage	*N*	Percentage	
Sex					
Male	6	43	7	50	
Female	8	57	7	50	
Ethnicity					
White	14	100	14	100	
Employment status					
Employed	9	64	6	43	
Retired	4	29	8	57	
Homemaker	1	7	0	0	
Smoking status					
Never-smoker	12	86	10	71	
Smoker	0	0	1	7	
Ex-smoker	2	14	3	22	
BMI category					
Healthy (<25 kg/m^2^)	6	43	4	28	
Overweight (25–29.9 kg/m^2^)	6	43	5	36	
Obese (>30 kg/m^2^)	2	14	5	36	

Independent T-tests report no difference between the exercise and control groups for baseline participant characteristics. Data are mean ± SD. MVPA, moderate + vigorous device measured physical activity level.

### Intervention adherence, compliance, and safety

Attendance at the online supervised exercise sessions was 92 ± 8%, and participation in home-based walks was 79 ± 14%. The prescribed exercise duration was achieved in 99 ± 23% of online supervised cycling sessions, and the prescribed exercise intensity was achieved in 100 ± 0% of online supervised cycling sessions. Therefore, the online supervised aerobic cycling program used in this study, which had a personalised progressive prescription of workload intensity, was feasible in treatment-naïve CLL, as >70% of participants completed >75% of sessions at the prescribed intensity (12). Supervised resistance exercise was progressive, and compliance to the prescribed repetitions was achieved in 100 ± 0% of online supervised sessions (**Table 2**). Participants complied to the duration of the home-based walk with a mean duration of 99 ± 23% (prescription = 45 min); however, participants did not comply to the intensity as only 62% of participants performed the home-based walks within the prescribed intensity of RPE 12-13/20 for 75% of the sessions (**Table 2**). One serious adverse event was reported (hospitalisation for pneumonia) that was unrelated to the trial, and one adverse event was reported (syncope following exercise) that was related to the trial.

**Table 2:** Exercise intervention compliance.

Prescribed intensity	Actual intensity completed	Below target (%)	Compliant (%)	Above target (%)	Participants compliant for >75% of sessions (%)
Supervised aerobic exercise					
Workload at −5% of AT (W)	55 ± 12	0 ± 0	100 ± 0	0 ± 0	100
Workload at AT (W)	58 ± 13	0 ± 0	100 ± 0	0 ± 0	100
Workload at +5% of AT (W)	61 ± 14	0 ± 0	100 ± 0	0 ± 0	100
Workload at +15% of AT (W)	67 ± 15	0 ± 0	100 ± 0	0 ± 0	100
Supervised resistance exercise					
20 reps	20 ± 0	0 ± 0	100 ± 0	0 ± 0	100
24 reps	24 ± 0	0 ± 0	100 ± 0	0 ± 0	100
30 reps	30 ± 0	0 ± 0	100 ± 0	0 ± 0	100
40 reps	40 ± 0	0 ± 0	100 ± 0	0 ± 0	100
Home-based walking					
RPE 12–13	12 ± 1	18 ± 30	71 ± 28	11 ± 15	62

Data are mean ± SD. Actual intensity was the workload in watts (W) prescribed for each participant using the baseline anaerobic threshold test (supervised aerobic exercise), number of repetitions completed according to exercise record card completed by researcher (supervised resistance exercise), and RPE score reported by participants in exercise diaries (home-based walking). Compliant = within prescribed range; below target = lower intensity than prescribed range; above target = higher intensity than prescribed range. AT, anaerobic threshold; Reps, repetitions; RPE, rating of perceived exertion.

### Physiological measurements

#### Body composition

The effects of intervention on body composition are reported in **Table 3A**. The exercise intervention elicited a 2% increase in DEXA-derived lean mass and a 4% decrease in gynoid fat percentage in the exercise group compared with a 0.4% decrease in lean mass and a 0.1% decrease in gynoid fat percentage in the control group (*p* < 0.01). DEXA-derived total body fat percentage decreased by 4% and 1% and fat mass decreased by 3% and 2% (*p* < 0.05), respectively, in the exercise and control groups, but there was no statistically significant difference between the groups (*p* > 0.05). Additionally, no statistically significant changes were observed for whole-body mass, android fat percentage, BMI, or bone mineral density (all *p* > 0.05).

**Table 3A:** Intervention-related changes to body composition and resting cardiovascular measurements.

	Group	Time point			Analysis		
		Pre-intervention	Post-intervention	% Δ	Interaction (time × group)	Main effect time	Main effect group
Body composition							
Body mass (kg)	Exercise Control	72.5 ± 17.4 82.6 ± 17.5	72.7 ± 18.4 81.7 ± 16.5	+ 0.3 − 1.1	F = 2.2, p = .16, partial η^2^ = .09	F = 1.1, p = .31, partial η^2^ = .05	F = 1.8, p = .20, partial η^2^ = .08
Body mass index (kg/m^2^)	Exercise Control	25.4 ± 5.2 28.3 ± 4.8	25.5 ± 5.6 28.1 ± 4.7	+ 0.5 − 0.9	F = 1.8, p = .19, partial η^2^ = .08	F = .30, p = .59, partial η^2^ = .01	F = 1.7, p = .20, partial η^2^ = .07
Body fat percentage (%)	Exercise Control	33.5 ± 7.6 35.0 ± 8.7	32.1 ± 7.4^##^ 34.5 ± 8.5^##^	− 4 − 1	F = 2.2, p = .15, partial η^2^ = .09	**F = 10.7, p = .004, partial η^2^ = .33**	F = .35, p = .56, partial η^2^ = .02
Android fat percentage (%)	Exercise Control	34.8 ± 9.5 39.4 ± 8.2	33.1 ± 9.5 38.8 ± 7.6	− 5 − 2	F = 0.9, p = .35, partial η^2^ = 0.04	F = 4.1, p = .056, partial η^2^ = 0.16	F = 2.1, p = .16, partial η^2^ = .09
Gynoid fat percentage (%)	Exercise Control	36.4 ± 8.6 35.8 ± 7.6	34.8 ± 8.5^***##^ 35.8 ± 7.9^***##^	− 4 − 0.1	**F = 8.8, p = .007, partial η^2^ = 0.29**	**F = 9.2, p = .006, partial η^2^ = 0.30**	F = .01, p = .94, partial η^2^ = .00
Fat mass (kg)	Exercise Control	24.5 ± 8.8 29.2 ± 10.4	23.7 ± 9.0^#^ 28.5 ± 9.9^#^	− 3 − 2	F = .05, p = .83, partial η^2^ = .002	**F = 5.8, p = .03, partial η^2^ = .21**	F = 1.5, p = .24, partial η^2^ = .06
Lean mass (kg)	Exercise Control	48.0 ± 11.5 53.4 ± 12	49.0 ± 11.9^**^ 53.2 ± 11.3^**^	+ 2 − 0.4	**F = 8.7, p = .01, partial η^2^ = .28**	F = 3.5, p = .07, partial η^2^ = .14	F = .99, p = .33, partial η^2^ = .04
Bone mineral density (g/cm^2^)	Exercise Control	1.14 ± 0.14 1.24 ± 0.14	1.14 ± 0.16 1.22 ± 0.13	+ 0.03 − 1.6	F = 3.05, p = .09, partial η^2^ = .12	F = 2.9, p = .11, partial η^2^ = .12	F = 2.8, p = .11, partial η^2^ = .11
Resting cardiovascular measurements							
Systolic blood pressure (mmHg)	Exercise Control	136 ± 19 138 ± 12	129 ± 15^#^ 130 ± 15^#^	− 5 − 6	F = .08, p = .78, partial η^2^ = .004	**F = 7.3, p = .01, partial η^2^ = .25**	F = .08, p = .78, partial η^2^ = .004
Diastolic blood pressure (mmHg)	Exercise Control	84 ± 9 87 ± 5	82 ± 6^# ^ 80 ± 7^#^	− 2 − 7	F = 2.3, p = .14, partial η^2^ = .10	**F = 7.4, p = .01, partial η^2^ = .25**	F = .19, p = .67, partial η^2^ = .01
Resting heart rate (beats/min)	Exercise Control	65 ± 12 62 ± 8	64 ± 12 61 ± 7	− 3 − 1	F = .21, p = .65, partial η^2^ = .01	F = 1.45, p = .24, partial η^2^ = .06	F = .72, p = .41, partial η^2^ = .03

Time × group analysis reports the interaction and main effects from two-way repeated measures ANOVA. Data are mean ± SD. ^**^indicates significant interaction between time and group, suggesting the difference from pre-intervention is different between groups at p < 0.05, ^***^indicates significant interaction between time and group, suggesting the difference from pre-intervention is different between groups at p < 0.01, ^#^indicates significant main effect of time, suggesting there is a difference from pre-intervention in both groups, but no difference between groups at p < 0.05, ^##^indicates significant main effect of time, suggesting there is a difference from pre-intervention in both groups, but no difference between groups at p < 0.01. Degrees of freedom were F(1,22) in all. ANOVA, analysis of variance; CLL, chronic lymphocytic leukaemia.Values in bold indicate the significant F-test statistics.

#### Resting cardiovascular measurements

The effects of intervention on resting cardiovascular measurements are reported in **Table 3A**. Resting systolic and diastolic blood pressure was lower at post-intervention in both groups (*p* < 0.05); the exercise group reduced systolic blood pressure by 5% from 136 ± 19 to 129 ± 15 mmHg and diastolic blood pressure by 2% from 84 ± 9 to 82 ± 6 mmHg, and the control group reduced systolic blood pressure by 6% from 138 ± 12 to 130 ± 15 mmHg and diastolic blood pressure by 7% from 87 ± 5 to 80 ± 7 mmHg, but there was no significant difference between groups (*p* > 0.05). No significant differences in resting heart rate were observed between conditions or over time (*p* > 0.05).

#### Cardiorespiratory fitness

The effects of intervention on cardiorespiratory fitness are reported in **Table 3B**. Heart rate (HR in bpm) and percentage of heart rate max (%HRmax) at which participants reached the anaerobic threshold decreased in both groups (*p* < 0.05). In the exercise group, the HR at which the participants reached the anaerobic threshold decreased from 110 ± 11 to 99 ± 16 bpm and %HRmax decreased from 69 ± 8 to 62 ± 11, and in the control group, the HR at which the participants reached the anaerobic threshold decreased from 107 ± 23 to 98 ± 17 bpm and %HRmax decreased from 69 ± 8 to 62 ± 11, but there was no difference between the groups. No change was observed within or between groups for the anaerobic threshold expressed as either mL·kg^−1^·min^−1^ or mL·lean mass kg^−1^·min^−1^ or the power (W) at which participants reached anaerobic threshold (all *p* > 0.05).

**Table 3B:** Intervention-related changes to cardiorespiratory and functional fitness measurements.

	Group	Time point			Analysis		
		Pre-intervention	Post-intervention	% Δ	Interaction (time × group)	Main effect time	Main effect group
Cardiorespiratory fitness							
Anaerobic threshold (mL·kg^−1^·min^−1^)	Exercise Control	14.2 ± 2.3 13.2 ± 3.5	14.1 ± 2.3 12.7 ± 4.7	− 1 − 4	F = .28, p = .60, partial η^2^ = .01	F = .64, p = .43, partial η^2^ = .03	F = .88, p = .36, partial η^2^ = .04
Anaerobic threshold (mL·lean mass kg^−1^·min^−1^)	Exercise Control	21.4 ± 2.9 19.7 ± 4.1	20.9 ± 3.2 18.5 ± 5.6	− 3 − 6	F = 1.0, p = .33, partial η^2^ = .05	F = 2.1, p = .16, partial η^2^ = .09	F = 1.6, p = .23, partial η^2^ = .07
Anaerobic threshold (W)	Exercise Control	58.5 ± 12.5 60.5 ± 24.1	62.5 ± 11.7 61.8 ± 25.7	+ 7 + 2	F = 1.0, p = .33, partial η^2^ = .05	F = 4.0, p = .06, partial η^2^ = .16	F = .01, p = .94, partial η^2^ = .00
Anaerobic threshold (HR bpm)	Exercise Control	110 ± 11 107 ± 23	99 ± 16^# ^ 98 ± 17^#^	− 11 − 9	F = .114, p = .74, partial η^2^ = .01	**F = 9.5, p = .01, partial η^2^ = .31**	F = .12, p = .74, partial η^2^ = .01
Anaerobic threshold (%HR_max_)	Exercise Control	69 ± 8 67 ± 15	62 ± 11^## ^ 60 ± 10^##^	− 10 − 11	F = .003, p = .96, partial η^2^ = .00	**F = 11.6, p = .003, partial η^2^ = .36**	F = .36, p = .55, partial η^2^ = .02
Functional fitness							
8ft up-and-go (s)	Exercise Control	6.2 ± 1.8 5.7 ± 1.2	5.4 ± 1 5.5 ± 1.1	− 13 − 3	F = .83, p = .37, partial η^2^ = .04	F = 2.4, p = .13, partial η^2^ = .10	F = .34, p = .57, partial η^2^ = .02
Sit-to-stand (number in 30 s)	Exercise Control	14 ± 3 14 ± 5	16 ± 4^#^ 15 ± 5^#^	+ 13 + 8	F = .42, p = .52, partial η^2^ = .02	**F = 9.5, p = .01, partial η^2^ = .30**	F = 9.5, p = .70, partial η^2^ = .01
Grip strength (kg)							
Right	Exercise Control	27 ± 11 32 ± 12	27 ± 12 33 ± 11	− 0.1 + 3	F = .39, p = .54, partial η^2^ = .02	F = .33, p = .57, partial η^2^ = .02	F = .16, p = .27, partial η^2^ = .06
Left	Exercise Control	26 ± 10 30 ± 11	26 ± 10 32 ± 11	− 1 + 7	F = 2.3, p = .14, partial η^2^ = .095	F = 1.6, p = .22, partial η^2^ = .07	F = 1.2, p = .28, partial η^2^ = .05
Upper-limb flexibility (cm)							
Right	Exercise Control	− 1 ± 9 − 4 ± 10	2 ± 8 − 4 ± 9	+ 247 + 7	F = 2.7, p = .12, partial η^2^ = .11	F = .63, p = .44, partial η^2^ = .03	F = .1.2, p = .30, partial η^2^ = .05
Left	Exercise Control	− 4 ± 7^$ ^ − 13 ± 12^$^	− 3 ± 9^$ ^ − 13 ± 13^$^	+ 27 − 1	F = .34, p = .57, partial η^2^ = .02	F = .29, p = .60, partial η^2^ = .01	**F = 5.1, p = .03, partial η^2^ = .20**
Lower-limb flexibility (cm)							
Right	Exercise Control	4 ± 7 − 3 ± 11	5 ± 7 0.1 ± 13	+ 21 + 102	F = 1.1, p = .30, partial η^2^ = .05	F = 3.1, p = .09, partial η^2^ = .13	F = 2.0, p = .17, partial η^2^ = .09
Left	Exercise Control	5 ± 6 − 2 ± 12	4 ± 8 − 1 ± 13	− 25 + 74	F = 1.5, p = .24, partial η^2^ = .06	F = .05, p = .83, partial η^2^ = .002	F = 2.0, p = .17, partial η^2^ = .08

Time × group analysis reports the interaction and main effects from two-way repeated measures ANOVA. Data are mean ± SD. ^#^indicates significant main effect of time, suggesting there is a difference from pre-intervention in both groups, but no difference between groups at p < 0.05, ^##^indicates significant main effect of time, suggesting there is a difference from pre-intervention in both groups, but no difference between groups at p < 0.01. Degrees of freedom were F(1,22) in all except for the cardiorespiratory and the upper-limb flexibility variables which were F(1,21) due to missing data from n = 1 control participant that did not complete the upper-limb flexibility tests or the anaerobic threshold test at post-intervention; therefore, the pre-intervention was removed from the analysis. Moreover, only complete datasets were analysed. ^$^ indicates significant main effect of group, suggesting that there is a difference between groups at pre- and post-intervention, but no difference over time at p < 0.05. Anaerobic threshold test is a submaximal exercise test used to measure cardiovascular fitness and presented here as millilitres of oxygen uptake per kilogram per minute and the power in watts at which the anaerobic threshold was achieved. ANOVA, analysis of variance; CLL, chronic lymphocytic leukaemia.Values in bold indicate the significant F-test statistics.

#### Functional fitness tests

The effects of intervention on functional fitness are reported in **Table 3B**. The number of sit-to-stand repetitions performed in 30 s increased in both groups (*p* < 0.05). The exercise group increased by 14% from 14 ± 3 to 16 ± 4 sit-to-stand repetitions, and the control group increased by 7% from 14 ± 5 to 15 ± 5 sit-to-stand repetitions, but there was no difference between the groups (**Table 3B**). No changes within or between groups were observed for time to complete the 8 Foot Up and Go Test, grip strength, upper-limb flexibility, and lower-limb flexibility (all *p* > 0.05).

#### Device measured physical activity

The effects of intervention on device-measured physical activity level are reported in **Supplementary Table 1**. Sedentary time, measured in minutes per week, reduced by 131 min from 713 ± 144 to 582 ± 309 in the exercise group, whereas the sedentary time increased by 32 min from 736 ± 91 to 768 ± 77 in the control group (*p* < 0.05) from pre intervention to post intervention. Vigorous activity decreased by 2.8 min per week from 3.9 ± 5.6 to 1.1 ± 2.2 in the exercise group, whereas the vigorous activity increased by 0.9 min from 0.5 ± 1.2 to 1.4 ± 2.6 in the control group (*p* < 0.05) from pre intervention to post intervention. No changes within or between groups were observed for the minutes per week of light, moderate, or MVPA (moderate to vigorous) activity (all *p* > 0.05).

#### Quality of life

The effects of intervention on Quality of life are reported in **Supplementary Tables 2A**–**2C**. No change was observed within or between the groups for any of the sub-scores of the European Organisation for Research and Treatment of Cancer Quality of Life Questionnaire C30 (EORTC QLQ-C30) (**Supplementary Table 2A**) and the CLL-specific module (EORTC QLQ-CLL16) (**Supplementary Table 2B** (all *p* > 0.05).

No change within or between the groups was observed for FACIT-fatigue, PSQI sleep quality, life satisfaction, or perceived stress (**Supplementary Table 2C**) (all *p* > 0.05).

### Blood immunophenotype

#### Full blood counts

As shown in **Table 4**, no main effects of time, from pre- to post-intervention, or interaction effects of time by group (Exercise vs. Control) were observed for total white blood cell count, haemoglobin (Hb), haematocrit (HCT), platelet count or neutrophil count (p > 0.05). As also shown in **Table 4**, a significant main effect of time was observed in the total red blood cell count, the main effect of time observed was a reduction in the frequency of red blood cells from pre- to post-intervention in both the exercise and control groups. In particular, the red blood cell count decreased from pre- to post- intervention by 0.3 ± 0.3 in the exercise group and 0.1 ± 0.3 in the control group (p = 0.01, partial η^2^ = 0.25).

**Table 4:** Intervention-related changes to haematological and leukocyte variables.

	Group	Time point		Absolute Δ from pre-intervention	Time × group analysis		
		Pre-intervention	Post-intervention		Interaction (time × group)	Main effect (time)	Main effect (group)
Haematological variables							
Total white blood cell count (cells ×10^9^/L)	Exercise Control	40.9 ± 25.0 50.3 ± 63.8	42.1 ± 25.3 49.9 ± 56.0	+ 1 ± 8 − 3 ± 10	F = .13, p = .73, partial η^2^ = .006	F = .04, p = .85, partial η^2^ = .002	F = .20, p = .66, partial η^2^ = .009
Red blood cell count (cells × 10^12^/L)	Exercise Control	4.5 ± 0.3 4.5 ± 0.5	4.2 ± 0.3^#^ 4.3 ± 0.4^#^	− 0.3 ± 0.3 − 0.1 ± 0.3	F = 1.2, p = .29, partial η^2^ = .05	**F = 7.5, p = .01, partial η^2^ = .25**	F = .22, p = .65, partial η^2^ = .01
Hb (g/L)	Exercise Control	133 ± 9 134 ± 11	129 ± 8 134 ± 12	− 5 ± 6 + 0.1 ± 6	F = 4.0, p = .06, partial η^2^ = .16	F = 4.1, p = .06, partial η^2^ = .16	F = .60, p = .45, partial η^2^ = .03
HCT (L/L)	Exercise Control	0.39 ± 0.05 0.40 ± 0.04	0.39 ± 0.03 0.40 ± 0.03	− 0.01 ± 0.1 − 0.01 ± 0.03	F = .001, p = .98, partial η^2^ = .00	F = .51, p = .48, partial η^2^ = .02	F = .48, p = .49, partial η^2^ = .02
Platelets (× 10^9^/L)	Exercise Control	194.3 ± 42.6 224.9 ± 87.3	176.7 ± 34.7 163.2 ± 92.5	− 18 ± 28 − 69 ± 137	F = 1.3, p = .27, partial η^2^ = .06	F = 4.1, p = .06, partial η^2^ = .16	F = .16, p = .70, partial η^2^ = .01
Neutrophils (cells × 10^6^/mL)	Exercise Control	3.3 ± 1.0 4.0 ± 1.4	3.5 ± 1.3 3.7 ± 1.1	+ 0.2 ± 1 − 0.4 ± 1	F = 4.4, p = .05, partial η^2^ = .17	F = .12, p = .74, partial η^2^ = .005	F = .81, p = .38, partial η^2^ = .04

Time × group analysis reports the interaction and main effects from two-way repeated measures ANOVA. Data are mean ± SD. Degrees of freedom were F(1,22) in all. The absolute change from Pre-intervention has been calculated using the following equation (mean post-intervention value − mean pre-intervention value).Values in bold indicate the significant F-test statistics.

#### B cells and B-CLL cells

As shown in **Table 5** and **Supplementary Figure 5**, no main effects of time or interaction effects of time by group were observed for total numbers of kappa or lambda clonally restricted B-CLL (CD3^−^/CD5^+^ CD19^+^) cells, CD38^+^ clonally restricted B-CLL cells, or CD49d^+^ clonally restricted B-CLL cells (all *p* > 0.05). In addition, no main effects of time, from pre- to post-intervention, or interaction effects of time by group (Exercise vs. Control) were observed for the absolute number of CD3^−^/CD5^−^ CD19^+^ polyclonal B cells (*p* > 0.05).

**Table 5:** Intervention-related changes to numbers of polyclonal B-cells (CD3^-^/CD5^-^ CD19^+^) and kappa or lambda clonally restricted B-CLL (CD3^−^/CD5^+^ CD19^+^) cells.

	Group	Time point		Absolute Δ from pre-intervention	Time × group analysis		
		Pre-intervention	Post-intervention		Interaction (time × group)	Main effect (time)	Main effect (group)
Cell frequency (cells/μL)							
Polyclonal B-cells (CD3^-^/CD5^−^ CD19^+^)	Exercise Control	72 ± 69 57 ± 48	54 ± 49 57 ± 67	− 18 ± 47 + 0.07 ± 31	F = 1.3, p = .27, partial η^2^ = .06	F = 1.3, p = .27, partial η^2^ = .06	F = .08, p = .78, partial η^2^ = .003
Clonal B-CLL cells (CD3^-^/CD5^+^ CD19^+^)	Exercise Control	28,795 ± 21,071 35,477 ± 57,005	25,375 ± 18,156 35,734 ± 50,815	− 3,421 ± 14,143 + 257 ± 13,146	F = .44, p = .52, partial η^2^ = .02	F = .32, p = .58, partial η^2^ = .01	F = .25, p = .62, partial η^2^ = .01
Clonal B-CLL CD38^+^	Exercise Control	22,310 ± 17,021 23,666 ± 34,394	22,152 ± 15,046 26,382 ± 39,684	− 158 ± 13,754 + 2,715 ± 12,300	F = .29, p = .59, partial η^2^ = .01	F = .23, p = .66, partial η^2^ = .01	F = .06, p = .82, partial η^2^ = .003
Clonal B-CLL CD49d^+^	Exercise Control	18,314 ± 18,569 14,810 ± 17,542	17,922 ± 16,005 18,923 ± 23,231	− 392 ± 8,757 + 4,113 ± 8,053	F = 1.7, p = .20, partial η^2^ = .07	F = 1.2, p = .29, partial η^2^ = .05	F = .03, p = .87, partial η^2^ = .001

Time × group analysis reports the interaction and main effects from two-way repeated measures ANOVA. Data are mean ± SD. Degrees of freedom were F(1,22) in all. The absolute change from pre-intervention has been calculated using the following equation (mean post-intervention value − mean pre-intervention value).

#### T cells

As shown in **Table 6A**, no main effects of time or interaction effects of time by group were observed for the frequency of CD3^+^ T cells (*p* > 0.05). As shown in **Table 6B**, no main effects of time or interaction effects of time by group were observed for total CD8^+^ T-cell frequency, or the frequency of CD8^+^ naïve T cells (T_NA_, CD45RA^+^CD27^+^), CD8^+^ central memory (T_CM_, CD45RA^−^CD27^+^), effector memory (T_EM_, CD45RA^−^CD27^−^), or CD8^+^ terminally differentiated effector memory cells re-expressing CD45RA^+^ (T_EMRA_, CD45RA^+^CD27^−^) (all *p* > 0.05). In addition, no main effects of time or interaction effects of time by group were observed for the frequency of CD8^+^ stem cell-like T cells (T_SCM_, CD95^+^CD127^+^), PD1^+^CD8^+^T_SCM_, PD1^+^CD8^+^, or PD1^+^TIM3^+^CD8^+^ T cells (all p > 0.05).

**Table 6A:** Intervention-related changes to numbers of total CD3^+^ and CD4^+^ T cells.

	Group	Time point		Absolute Δ from pre-intervention	Time × group analysis		
		Pre-intervention	Post-intervention		Interaction (time × group)	Main effect (time)	Main effect (group)
T-cell frequency (cells/μL)							
CD3^+^	Exercise Control	1,837 ± 539 1,777 ± 714	1,616 ± 1,114 1,259 ± 690	− 221 ± 1,100 − 517 ± 557	F = .73, p = .40, partial η^2^ = .03	F = 4.5, p = .05, partial η^2^ = .17	F = .59, p = .45, partial η^2^ = .03
CD4 T-cell frequencies (cells/μL)							
CD3^+^CD4^+^	Exercise Control	1,081 ± 258 1,068 ± 346	907 ± 400^##^ 749 ± 376^##^	− 174 ± 415 − 320 ± 334	F = .91, p = .35, partial η^2^ = .04	**F = 10.4, p = .004, partial η^2^ = .32**	F = .49, p = .49, partial η^2^ = .04
T_NA_	Exercise Control	587 ± 179 663 ± 225	473 ± 191^##^ 403 ± 228^##^	− 114 ± 181 − 230 ± 207	F = 2.1, p = .16, partial η^2^ = .09	**F = 18.6, p = <.001, partial η^2^ = .46**	F = .03, p = .88, partial η^2^ = .001
T_SCM_	Exercise Control	148 ± 114 142 ± 83	101 ± 44^#^ 81± 52^#^	− 48 ± 122 − 61 ± 56	F = .13, p = .72, partial η^2^ = .01	**F = 8.2, p = .01, partial η^2^ = .27**	F = .26, p = .62, partial η^2^ = .27
PD1^+^ T_SCM_	Exercise Control	34 ± 13 38 ± 29	27 ± 12^##^ 21± 19^##^	− 7 ± 13 − 17 ± 18	F = 2.6, p = .12, partial η^2^ = .11	**F = 13.4, p = .001, partial η^2^ = .38**	F = .02, p = .89, partial η^2^ = .001
T_CM_	Exercise Control	387 ± 226 337 ± 155	365 ± 293 269 ± 155	− 22 ± 232 − 68 ± 126	F = .38, p = .55, partial η^2^ = .02	F = 1.5, p = .24, partial η^2^ = .06	F = .88, p = .34, partial η^2^ = .04
T_EM_	Exercise Control	81 ± 105 67 ± 72	53 ± 47 53 ± 45	− 28 ± 66 − 15 ± 39	F = .38, p = .54, partial η^2^ = .02	F = 3.9, p = .06, partial η^2^ = .15	F = .06, p = .80, partial η^2^ = .003
T_EMRA_	Exercise Control	26 ± 23 31 ± 43	17 ± 16^##^ 24 ± 37^##^	− 9 ± 10 − 7 ± 10	F = .29, p = .59, partial η^2^ = .01	**F = 13.5, p = .001, partial η^2^ = .38**	F = .24, p = .63, partial η^2^ = .011
PD1^+^	Exercise Control	371 ± 179 330 ± 166	334 ± 241 248 ± 162	− 37 ± 225 − 82 ± 135	F = .37, p = .55, partial η^2^ = .02	F = 2.5, p = .13, partial η^2^ = .10	F = .89, p = .36, partial η^2^ = .04
PD1^+^TIM3^+^	Exercise Control	18 ± 17 18 ± 12	27 ± 37 16 ± 11	+ 10 ± 34 − 2 ± 16	F = 1.2, p = .28, partial η^2^ = .05	F = .50, p = .49, partial η^2^ = .02	F = .69, p = .42, partial η^2^ = .03
CTLA4^+^	Exercise Control	16 ± 17 18 ± 20	14 ± 15 14 ± 14	− 2 ± 17 − 5 ± 26	F = .10, p = .75, partial η^2^ = .03	F = .61, p = .44, partial η^2^ = .03	F = .10, p = .75, partial η^2^ = .04
FOXP3^+^ T_REGS_	Exercise Control	61 ± 25 62 ± 37	53 ± 23 56 ± 56	− 8 ± 17 − 7 ± 27	F = .01, p = .91, partial η^2^ = .001	F = 2.4, p = .14, partial η^2^ = .01	F = .01, p = .91, partial η^2^ = .001
CTLA4^+^ FOXP3^+^ T_REGS_	Exercise Control	45 ± 22 36 ± 10	38 ± 23 31 ± 28	− 7 ± 19 − 5 ± 26	F = .02, p = .88, partial η^2^ = .001	F = .21, p = .21, partial η^2^ = .07	F = 1.2, p = .29, partial η^2^ = .05

Time × group analysis reports the interaction and main effects from two-way repeated measures ANOVA. Data are mean ± SD. ^#^indicates significant main effect of time, suggesting there is a difference from pre-intervention in both groups, but no difference between groups at p < 0.05, ^##^indicates significant main effect of time, suggesting there is a difference from pre-intervention in both groups, but no difference between groups at p < 0.01. Degrees of freedom were F(1,22) in all except CD4 CTLA4 which was F(1,21) due to a problem with one of the control participants post-intervention sample; therefore, the pre-intervention sample was also removed. The absolute change from pre-intervention has been calculated using the following equation (mean post-intervention value − mean pre-intervention value).Values in bold indicate the significant F-test statistics.

**Table 6B:** Intervention-related changes to numbers of total CD8^+^ T cells.

	Group	Time point		Absolute Δ from pre-intervention	Time × group analysis		
		Pre-intervention	Post-intervention		Interaction (time × group)	Main effect (time)	Main effect (group)
CD8 T-cell frequency (cells/μL)							
CD3^+^CD8^+^	Exercise Control	585 ± 236 590 ± 328	574 ± 679 428 ± 285	− 10 ± 678 − 162 ± 209	F = .59, p = .45, partial η^2^ = .03	F = .76, p = .39, partial η^2^ = .03	F = .27, p = .61, partial η^2^ = .01
T_NA_	Exercise Control	317 ± 128 331 ± 180	288 ± 310 253 ± 201	− 30 ± 315 − 79 ± 89	F = .29, p = .60, partial η^2^ = .01	F = 1.4, p = .25, partial η^2^ = .06	F = .02, p = .89, partial η^2^ = .001
T_SCM_	Exercise Control	117 ± 56 159 ± 126	111 ± 82 126 ± 127	− 7 ± 79 − 33 ± 55	F = .48, p = .49, partial η^2^ = .02	F = 2.4, p = .14, partial η^2^ = .10	F = .40, p = .53, partial η^2^ = .02
PD1^+^ T_SCM_	Exercise Control	64 ± 38 72 ± 67	59 ± 56 58 ± 79	+ 0.04 ± 55 − 14 ± 36	F = .22, p = .64, partial η^2^ = .01	F = .82, p = .38, partial η^2^ = .04	F = .02, p = .89, partial η^2^ = .001
T_CM_	Exercise Control	127 ± 80 99 ± 76	146 ± 203 86 ± 66	+ 19 ± 183 − 13 ± 45	F = .38, p = .54, partial η^2^ = .02	F = .02, p = .90, partial η^2^ = .001	F = 1.2, p = .28, partial η^2^ = .05
T_EM_	Exercise Control	28 ± 35 23 ± 27	28 ± 43 19 ± 23	+ 0.14 ± 49 − 4 ± 9	F = .09, p = .77, partial η^2^ = .004	F = .07, p = .79, partial η^2^ = .003	F = .42, p = .53, partial η^2^ = .02
T_EMRA_	Exercise Control	113 ± 94 137 ± 174	112 ± 150 70 ± 58	− 0.3 ± 149 − 66 ± 128	F = 1.4, p = .26, partial η^2^ = .06	F = 1.4, p = .25, partial η^2^ = .06	F = .04, p = .84, partial η^2^ = .06
PD1^+^	Exercise Control	262 ± 138 213 ± 145	276 ± 403 174 ± 170	+ 14 ± 407 − 39 ± 77	F = .21, p = .65, partial η^2^ = .01	F = .05, p = .83, partial η^2^ = .002	F = .96, p = .34, partial η^2^ = .04
PD1^+^TIM3^+^	Exercise Control	22 ± 25 21 ± 18	52 ± 101 18 ± 16	+ 31 ± 92 − 3 ± 14	F = 1.7, p = .21, partial η^2^ = .07	F = 1.1, p = .30, partial η^2^ = .05	F = 1.2, p = .29, partial η^2^ = .05
CD4: CD8 ratio	Exercise Control	2.0 ± 0.6 2.4 ± 1.5	2.1 ± 0.6 2.5 ± 1.7	+ 0.1 ± 1 + 0.1 ± 0.4	F = .01, p = .91, partial η^2^ = .001	F = .78, p = .39, partial η^2^ = .03	F = .50, p = .49, partial η^2^ = .02

Time × group analysis reports the interaction and main effects from two-way repeated measures ANOVA. Data are mean ± SD. Degrees of freedom were F(1,22) in all. The absolute change from pre-intervention has been calculated using the following equation (mean post-intervention value − mean pre-intervention value).

As also shown in **Table 6A**, a significant main effect of time was observed in the total CD4^+^ count and various CD4^+^ subsets, and the main effect of time observed was a reduction in the frequency of cells from pre- to post-intervention in both the exercise and control groups. In particular, the CD4^+^ count decreased from pre- to post-intervention by 173 ± 416 in the exercise group and 319 ± 334 in the control group (*p* = 0.004, partial η^2^ = 0.32), the frequency of CD4^+^T_NA_ decreased from pre- to post-intervention by 114 ± 181 in the exercise group and 231 ± 207 in the control group (*p* = 0.001, partial η^2^ = 0.46), CD4^+^T_SCM_ decreased from pre- to post-intervention by 47 ± in the exercise group and 61 ± in the control group (*p* = 0.01, partial η^2^ = 0.27), and CD4^+^PD1^+^T_SCM_ decreased from pre- to post-intervention by 7 ± 13 in the exercise group and 17 ± 18 in the control group (*p* = 0.001, partial η^2^ = 0.38), and CD4^+^T_EMRA_ decreased from pre- to post-intervention by 9 ± 11 in the exercise group and 7 ± 10 in the control group (*p* = 0.001, partial η^2^ = 0.38). No main effects of time or interaction effects of time by group were observed in CD4^+^T_CM_, CD4^+^T_EM_, CD4^+^PD1^+^, CD4^+^PD1^+^TIM3^+^, CD4^+^CTLA4^+^, CD4^+^FOXP3^+^ T_REGS_ or CD4^+^CTLA4^+^FOXP3^+^ T_REGS_ populations (all *p* > 0.05).

## Discussion

The primary finding of this study was that 16 weeks of personalised, progressive, home-based exercise training—consisting of supervised aerobic and resistance exercise, with an additional unsupervised walk—was feasible in people with treatment-naïve CLL who passed pre-trial screening, and we preliminarily conclude the exercise program was safe. The aerobic and resistance components of the exercise intervention were deemed feasible as >70% of participants completed >75% of sessions at the prescribed intensity (12). Participants complied to the duration of the unsupervised walk; however, participants did not comply to the intensity as only 62% of participants performed the home-based walks within the prescribed intensity for 75% of the sessions. Emery et al. observed a similar finding during a 16-week exercise intervention in 15 participants diagnosed with the asymptomatic precursor conditions to multiple myeloma (smouldering multiple myeloma (SMM) and monoclonal gammopathy of undetermined significance (MGUS)). In that study, participants exceeded the prescribed duration of the unsupervised walk, but most of the walks were below the prescribed intensity; 13% of participants performed the home-based walks within the prescribed intensity for 75% of the sessions (11). In addition, telephone coaching throughout a home-based physical activity intervention in patients with CLL was deemed feasible whereas email coaching did not meet the feasibility criteria (18). Together, these findings highlight the importance of supervision to achieve the prescribed exercise intensity. Throughout the study, one serious adverse event, which was unrelated to the trial, and one adverse event, which was related to the trial, were reported. This finding suggests that preliminarily we can conclude that the exercise program used in this study was safe. Finally, this pilot trial highlights the importance of a resting ECG pre-screening measure in this patient population, as 22.5% of participants (*n* = 9) were excluded due to an undiagnosed cardiac abnormality.

A key secondary outcome of this study was to preliminarily assess the effects of exercise training on the absolute numbers of blood CLL cells, measured as CD5^+^/CD19^+^ with light-chain restriction. We found that disease activity did not differ between the exercise and control groups over the 16 weeks. This implies that, in the timeframe of the intervention used in this trial, exercise training did not reduce disease outgrowth measured by absolute B-CLL cell counts. Our findings contrast those of a smaller non-randomised pilot study in treatment-naïve CLL which reported 21.4% less disease outgrowth of CD5^+^/CD19^+^ B-CLL cells in an exercise group compared with a control group following 12 weeks of HIIT (12). Nevertheless, the findings of our study could be considered unsurprising. The anticancer effects of physical activity appear to be largely dictated by exercise-induced alterations to anticancer immunity, which relies on tumour cells exhibiting immunogenic features in order for the anticancer immunological effects of exercise to materialise—as reviewed elsewhere (10). However, treatment-naïve CLL is typically considered a poorly immunogenic cancer, and thus, it might be expected that any exercise-induced changes to immune function may be redundant if the CLL tumour cell clones are non-immunogenic (36). This concept is supported by epidemiological observations showing that lymphocytic leukaemia, of which CLL comprises a large proportion of cases, does not show reduced incidence among highly active people (9), thus implying that anti-tumour effects of exercise might not manifest in treatment-naive CLL. The finding herein that 16 weeks of progressive exercise training did not suppress CLL disease activity also aligns with findings from exercise studies during active surveillance in prostate cancer (16, 17). For example, it was shown that an exercise intervention lasting 12 months during active monitoring for prostate cancer showed no change in prostate-specific antigen (PSA) (17), a marker of disease activity. We note that, like CLL, early-stage prostate cancer is poorly immunogenic and does not show reduced incidence among highly active people (9), and so the anti-tumour benefits of exercise training may not manifest in that setting either.

In our study here, we extended our flow cytometric analyses of B-CLL cells to include CD38 and CD49d, which are each considered prognostic markers for disease progression in treatment-naïve CLL (37–39). For example, CD49d^+^ CLL cells are associated with more aggressive disease, including higher proliferation rates and a different mutational landscape (40). The findings from our study show that the exercise group appeared to have maintenance of CD49d^+^ and CD38^+^ CLL cells in blood over time, respectively, whereas the control arm displayed a mean outgrowth of approximately 2,700 (CD38^+^) and 4,000 (CD49^+^) cells per microlitre, respectively. The interaction between intervention and groups did not reach statistical significance; however, a small (η^2^ = 0.01) and a moderate (η^2^ = 0.07) effect size was observed respectively for the CD38^+^ and CD49^+^ cells. This could represent a clinically meaningful difference given that the increase in CD38^+^ CLL cells was 12% in the control group over time and previous cohort data have demonstrated that patients who show a fluctuation of >5% in CD38 expression over time have a shorter time to treatment and overall survival than patients without the fluctuation in CD38 expression (41). It is important to note that the study herein was not designed—i.e., not statistically powered—for hypothesis testing, as is the nature of pilot studies (42, 43). Thus, it cannot be concluded from this study that exercise does—or does not—affect disease activity. Therefore, our findings regarding CD49d^+^ and CD38^+^ CLL cells warrant evaluation in a larger trial.

This pilot study also preliminarily assessed the effects of exercise training on body composition. Here, we show that 16 weeks of combined aerobic and resistance exercise training increased DEXA-derived total lean muscle mass by 2% in the exercise group with a reduction of 0.4% in the usual care control group. In addition, there was a 4% reduction in gynoid fat percentage in the exercise group with a 0.1% reduction in the usual care control group. The specific gynoid location of this body fat reduction is likely due to the high number of muscles recruited from the gynoid region during static cycling exercise (44). These results are consistent with those from Courneya and colleagues who observed a ~1.6% lean mass difference between the control and exercise group following 12-weeks of supervised aerobic training in haematologic cancer patients that included CLL patients (*n* = 14 of 122) (19). In addition, the results are consistent with those from MacDonald and colleagues who reported a 3.1% increase in bioelectrical impedance measured lean muscle mass, in an exercise group following 12 weeks of supervised, combined resistance and HIIT exercise in treatment-naïve CLL patients (*n* = 10 exercise, *n* = 6 controls) (12). The increase in lean mass observed by MacDonald and colleagues translated to improvements in maximal muscle strength in legs and upper body muscles in the exercise group compared with the control group. Similar results were observed by Furzer and colleagues who showed strength improvements following 12 weeks of supervised aerobic plus resistance exercise training in haematologic cancer patients recently completing treatment (*n* = 18 exercise, *n* = 19 controls) (45). Although the study herein did not assess maximal strength, we observed a small 1–2-repetition increase in the sit to stand test, a functional measure of strength, in both groups at the 16-week time point—together suggesting that the exercise group alone increased lean muscle mass, but both groups improved lower body strength and power. Exercise interventions that induce muscle hypertrophy are relevant to CLL given that cancer treatments and advanced age are catabolic for lean tissue (46). Furthermore, increases to muscle-derived cytokines may be involved in maintaining immune competence against immunogenic cancer cells to reduce disease outgrowth (10) and risk of secondary malignancy (10). Future studies should aim to determine whether increasing lean muscle mass and thus potentially influencing muscle-derived cytokines, maintains immune competence against mutated (i.e., more immunogenic) tumour cell clones, thus delaying time to treatment in CLL.

In this study, we observed no improvement in cardiorespiratory fitness measured by CPET within or between the groups following the 16-week exercise intervention. Previous studies in mixed haematological cancers have observed increases in cardiorespiratory fitness following a structured exercise intervention (19, 45); however, these studies recruited a mixture of patients on and off treatment. Therefore, the baseline fitness of participants was considerably lower than the study herein. In the study conducted by MacDonald and colleagues that consisted of 12 weeks of supervised, combined resistance and HIIT exercise in treatment-naïve CLL patients (12), both of the exercise and control groups improved their cardiorespiratory fitness, with the exercise group improving by 5.3%, whereas the control group improved by 10.3%. The authors suggested that this difference was the result of contamination in the control group, similar to the 12%–19% increase in the control group and 16%–25% increase in the exercise group observed by Persoon et al. following 18 weeks of HIIT in mixed haematological cancers following stem cell transplant (*n* = 54 exercise, *n* = 55 control) (47). A possible explanation for our finding of no change in cardiorespiratory fitness is that the exercise prescription was not sufficient to improve cardiorespiratory fitness. Alternatively, it may be that haematological conditions in CLL are unfavourable to yield gains in fitness over a short timeframe. For example, we note a reduction in red blood cell (RBC) counts over time (in both groups combined) from pre to post intervention, which may contribute to a limiting of oxygen carrying capacity of the blood, and thus preventing an improvement in cardiorespiratory fitness. Finally, we observed a reduction of 131 min per week in device measured sedentary time, in the exercise group following the intervention. Insufficient physical activity and high levels of sedentary behaviour are high-risk factors for cardiometabolic and non-communicable diseases (48). A recent, observational cohort study reported that 21.3% of the treatment naïve CLL patients included were inactive, measured using the Stanford Brief Activity Summary (SBAS) self-report questionnaire (49). Since low fitness and physical function are predictive of poor survival following treatment for CLL (50) and people living with CLL often have multiple cardiometabolic comorbidities (51), a reduction in sedentary time in this population could demonstrate a healthy behaviour change, potentially protecting against cardiometabolic conditions.

A consideration within the present study is that we show some evidence of ‘white coat hypertension’ during the pre-intervention visit (52). In particular, a small reduction in both resting systolic and diastolic blood pressure measurements were evident in both groups at the post-intervention time point. In the exercise group, the resting systolic blood pressure reduced by 5% and the resting diastolic reduced by 2%; in the control group, the resting systolic blood pressure reduced by 6% and the resting diastolic reduced by 7%. Additionally, it is possible that this ‘white coat hypertension’ could also be partly responsible for slightly higher numbers of CD4^+^ T cells and CD4^+^ T-cell subsets in both groups at the pre-intervention time point. T cells are highly responsive to both psychological and exercise stresses, and they are mobilised into the peripheral blood circulation via a mechanism that is partly dependent on catecholamine activation of lymphocyte β2 adrenergic receptors (AR) (53–55); therefore, the higher blood pressure observed in the pre-intervention visit could have resulted in the large effect (partial η^2^ = 0.17) of a higher number of resting CD3^+^ T cells observed in both groups at the pre-intervention time point. However, if this ‘white coat hypertension’ was indeed influencing cell counts at baseline, it is unclear why only CD4^+^ T cells were affected, but CD8^+^ T cells—which are more sensitive to adrenergic stimulation—were not. Moreover, declines in blood cell counts following exercise interventions are common in the literature. For example, MacDonald et al. showed a reduction of 10.4% in CD4^+^ T cells in treatment-naïve CLL patients (12). Crane and colleagues showed a lower percentage of HLA^−^DR^+^PD-1^+^CD4 T cells (13) following exercise training in CLL patients. Niemiro and colleagues observed a reduction in CD4^+^ T-cell numbers following a 12-week exercise high-intensity interval exercise intervention (*n* = 8) compared with a moderate-intensity exercise intervention (*n* = 8) and usual care (*n* = 8) in older women at high risk of breast cancer (56). Finally, Arana Echarri and colleagues observed lower counts and activation of CD4^+^ EMRA T cells following 8 weeks of exercise training in breast cancer survivors (*n* = 20) (57). Therefore, the relationships between CD4^+^ T cells and exercise in CLL warrant further investigation. Nevertheless, future exercise studies in CLL should include a comprehensive familiarisation session prior to the experimental trials to eliminate potential contamination from ‘white coat hypertension’.

A limitation of our study is the relatively short duration of the exercise intervention. The clinical course of CLL is heterogeneous with only 5% of patients diagnosed requiring immediate treatment (58); many of the remaining 95% patients with treatment-naïve CLL will live for decades without needing treatment (59). Therefore, the 16 weeks herein is likely not long enough to see clinically recognised changes in markers of disease progression in the control group, and in turn not long enough to see if exercise has any impact on disease progression in the exercise group. Additionally, while the aerobic exercise in the present study was individually prescribed, it may not have been sufficiently vigorous. There was no improvement in cardiorespiratory fitness in the exercise group, but there was also no reduction in compliance to the aerobic exercise as the intensity increased from moderate to vigorous, suggesting that perhaps the prescription of 15% above the participants’ anaerobic threshold was not vigorous enough and could have been higher. In addition, while the resistance prescription elicited an increase in muscle mass, this prescription could be optimised to further promote hypertrophy with the addition of weights and appropriate nutritional guidance. Finally, we observed a discrepancy between patient self-reported physical activity using the IPAQ, collected during the pre-screening telephone call, and the device-measured physical activity level measured immediately following visit 1. This resulted in group level MVPA measuring <150 min per week and thus suggesting physical inactivity overall in this cohort; however, n=4 participants in both the exercise and control groups measured >150 min of MVPA per week, which suggests those participants meet the physical activity guidelines. In future studies, the device-worn physical activity level should be used to confirm the self-report physical activity level (IPAQ) prior to inclusion in the trial.

A strength of this pilot study was the inclusion of participants of different ages and sex; however, a larger sample is required to include diversity in ethnicity.

In conclusion, an individually prescribed 16-week, home-based, progressive exercise intervention was feasible and safe in people with treatment-naïve CLL who passed pre-trial screening. No differences were observed for light-chain-restricted CD5^+^/CD19^+^ CLL cells between groups over time, which may be considered unsurprising given the poor immunogenicity of tumour cell clones in treatment-naïve CLL. Among other secondary outcomes measured, we observed improvements to lean (i.e., muscle) mass, and reductions to gynoid fat in exercisers compared with controls.

## Data Availability

The raw data supporting the conclusions of this article will be made available by the authors, without undue reservation.

## Supplementary material

Supplementary Figure 1 Representative B-CLL flow cytometry analysis with a sample from a trial participant. FSC versus SSC was plotted to identify Mononuclear cells (MNC) **(A)**. Singlets were gated to discard doublets **(B)**. Viable cells were identified **(C)**. CD45^+^ cells were identified **(D)**. CD3^+^ T-cells were discarded **(E)**. To identify B-CLL cells (CD19^+^ CD5^+^) and polyclonal B-cells (CD19^+^ CD5^−^) CD19 was gated against CD5 in the CD3^−^ population **(F)**. Within the polyclonal B-cell population kappa was gated against lambda to identify the polyclonal B-cells with restriction to either kappa or lambda **(G)**. The gate drawn in the polyclonal B-cell population was then copied to the B-CLL cell population to identify the clonally restricted B-CLL cells **(H)**. Prognostic markers CD49d^+^ **(I)** and CD38^+^ **(J)** were also identified in the clonally restricted B-CLL cells.

Supplementary Figure 2 Representative CD8^+^ T-cell flow cytometry analysis with a sample from a trial participant. FSC versus SSC was plotted to identify Mononuclear cells (MNC) **(A)**. Singlets were gated to discard doublets **(B)**. CD19^+^ B-cells were excluded **(C)**. CD3^+^ T cells were identified in the CD19^−^ population **(D)**. CD8^−^ CD4^+^ helper and CD8^+^ CD4^−^ cytotoxic cells were identified **(E)**. Subsets were gated using CD27 and CD45RA to discriminate by maturation/differentiation **(F)** into Naïve (NA) (CD27^+^ CD45RA^+^), Central Memory (CM) (CD27^+^ CD45RA^−^), Effector Memory (EM) (CD27^−^ CD45RA^−^) and Effector Memory expressing CD45RA (EMRA) (CD27^−^ CD45RA^+^). Exhausted T-cells were identified from the CD8^+^ T-cells by gating TIM3 against PD1 as both have been identified as markers of T-cell exhaustion **(G)**. TIM3^+^ **(H)** and PD1^+^ **(I)** exhausted T-cells were also identified within the total CD8^+^ T-cell population. CD8^+^ stem cell like T cells (TSCM) were identified by CD95 expression from the naïve T cell pools **(J)**, and CD95^+^ cells were gated against CD127, as both have been identified as markers of TSCM **(K)**. Exhausted T-cells were identified from the CD8^+^ TSCM population by gating TIM3 against PD1 as both have been identified as markers of T-cell exhaustion **(L)**. TIM3^+^ **(M)** and PD1^+^ **(N)** exhausted T-cells were also identified within the total CD8+ TSCM population.

Supplementary Figure 3 Representative CD4^+^ T-cell flow cytometry analysis with a sample from a trial participant. This figure follows **Supplementary Figure 2A–E** and shows the CD4^+^ T-cell gating strategy. Total CD4^+^ T-cell population was gated using CD27 and CD45RA to discriminate by maturation/differentiation **(A)** into Naïve (NA) (CD27^+^ CD45RA^+^), Central Memory (CM) (CD27^+^ CD45RA^−^), Effector Memory (EM) (CD27^−^ CD45RA^−^) and Effector Memory expressing CD45RA (EMRA) (CD27^−^ CD45RA^+^). Exhausted T-cells were identified from the CD4^+^ T-cells by gating TIM3 against PD1 as both have been identified as markers of T-cell exhaustion **(B)**. TIM3^+^ **(C)** and PD1^+^ **(D)** exhausted T-cells were also identified within the total CD4^+^ T-cell population. CTLA4 expression was identified in the total CD4^+^ T-cell population **(E)**. CD4^+^ stem cell like T cells (TSCM) were identified by CD95 expression from the naïve T cell pools **(F)**, and CD95^+^ cells were gated against CD127, as both have been identified as markers of TSCM **(G)**. Exhausted T-cells were identified from the CD4^+^ TSCM population by gating TIM3 against PD1 as both have been identified as markers of T-cell exhaustion **(H)**. TIM3^+^ **(I)** and PD1^+^ **(J)** exhausted T-cells were also identified within the total CD8^+^ TSCM population. T-regulatory cells were identified by first gating CD25^+^ FoxP3^+^ in the total CD4^+^ T-cell population **(K)** then gating the CD25^+^ FoxP3^+^ against CD127, to identify the CD25^+^ FoxP3^+^ CD127dim CD4 T-regulatory cells **(L)**. CTLA4 expression was identified in the T-regulatory cell population **(M)**.

Supplementary Figure 4 Schematic diagram showing the structure of supervised and unsupervised exercise sessions, for the 16-week exercise program. Each trial week comprised two supervised and one unsupervised home-based exercise session. Colour-coding is used to link the trial week to the prescribed exercise intensities: Lightest grey = weeks 1-2, wattage that equates to - 5% of ventilatory threshold, 2 sets of 10 repetitions; Lighter grey = weeks 3-6, wattage that equates to ventilatory threshold, 2 sets of 12 repetitions; Dark grey = weeks 7-10, wattage that equates to + 5% of ventilatory threshold, 3 sets of 10 repetitions; Darkest grey = weeks 11-16, wattage that equates to + 15% of ventilatory threshold, 4 sets of 10 repetitions. Supervised sessions commenced with a five-minute warm-up, followed by 30 minutes of cycling (8 minutes work:2 minutes active recovery). Next, the prescribed sets and repetitions of six standardised resistance exercises were performed, this was split into 2 sessions, session 1 was the first session of the week and 3 exercises were performed. Session 2 was the second session of the week and the next 3 exercises were performed. If participants experienced pain in given movement patterns, substitutions were offered, e.g., reverse fly ➔ low row, squat ➔ sit-to-stand. Home-based exercise was a 45-minute walk at moderate intensity. The total prescribed weekly volume of aerobic exercise was 100 minutes at moderate intensity in weeks 1-6 and 100 minutes at moderate-vigorous intensity in weeks 7-16. WU = Warm-up; AR = Active recovery; VT = Ventilatory threshold; RPE = Rating of perceived exertion.

Supplementary Figure 5 Pre- to post-intervention changes to CLL cells. Pre- to post-exercise training changes to clonally restricted CLL cells (exercise group **A**, control group **B**), clonally restricted CLL cells expressing CD49d^+^ (exercise group **C**, control group **D**), and clonally restricted CLL cells expressing CD38^+^ (exercise group **E**, control group **F**). Dashed lines show individual responses from pre to post intervention. Black circles show the individual change (Post – Pre) in exercise participants, grey circles show the individual change (Post – Pre) in control participants.
