# Supplementary material for: A 16-week progressive exercise training intervention in treatment-naïve chronic lymphocytic leukaemia: a randomised-controlled pilot study
Source: Front Oncol. 2024 Dec 5;14:1472551. doi: 10.3389/fonc.2024.1472551 (PMC11655450; doi:10.3389/fonc.2024.1472551)
Supplement: Supplementary file 1 [file Table1.docx]

| Supplementary Table 1. Intervention related changes to the device measured physical activity levels. | | | | | | | | | |
| --- | --- | --- | --- | --- | --- | --- | --- | --- | --- |
|  |  | Time point | | | |  | Time x Group Analysis | | |
|  | Group | Pre-Intervention |  | Post-Intervention | Δ |  | Interaction (time x group) | Main effect (time) | Main effect (group) |
| Sedentary (minutes/week) | Exercise Control | 713 ± 144 736 ± 91 |  | 582 ± 309^**^ 768 ± 77^**^ | −131 +32 |  | **F = 4.5, p = .04, partial η2 = .17** | F =1.6, p = .21, partial η2 = .07 | F =3.0, p = .10, partial η2 = .12 |
| Light activity (minutes/week) | Exercise Control | 184 ± 65 203 ± 108 |  | 120 ± 59 183 ± 37 | −65 −20 |  | F =1.1, p = .31, partial η2 = .05 | F =4.1, p = .06, partial η2 = .16 | F =3.7, p = .07, partial η2 = .15 |
| Moderate activity (minutes/week) | Exercise Control | 134 ± 61 118 ± 46 |  | 115 ± 82 122 ± 43 | −19 +4 |  | F =1.2, p = .30, partial η2 = .05 | F =.45, p = .51, partial η2 = .02 | F =.04, p = .85, partial η2 = .002 |
| Vigorous activity (minutes/week) | Exercise Control | 3.9 ± 5.6 0.5 ± 1.2 |  | 1.1 ± 2.2^**^ 1.4 ± 2.6^**^ | −2.8 +0.9 |  | **F = 6.2, p = .02, partial η2 = .22** | F =1.7, p = .20, partial η2 = .07 | F =2.0, p = .18, partial η2 = .08 |
| MVPA (minutes/week) | Exercise Control | 138 ± 64 118 ± 47 |  | 116 ± 83 124 ± 44 | −22 +6 |  | F =1.5, p = .23, partial η2 = .06 | F =.56, p = .46, partial η2 = .03 | F =.07, p = .80, partial η2 = .003 |

*Time x group analysis reports the interaction and main effects from two-way repeated measures ANOVA. Data are mean ± SD. ^**^indicates significant interaction between time and group, suggesting the difference from pre-intervention is different between groups at p < 0.05, Degrees of freedom were F(1,22) in all. ANOVA, analysis of variance; MVPA, moderate to vigorous activity (Moderate activity + Vigorous activity in minutes/week).*

| Supplemental Table 2A. Intervention related changes to the Quality of life (EORTC QLQ-C30) questionnaire. | | | | | | | | | |
| --- | --- | --- | --- | --- | --- | --- | --- | --- | --- |
|  |  | Time point | | | |  | Time x Group Analysis | | |
|  | Group | Pre-Intervention |  | Post-Intervention | Δ |  | Interaction (time x group) | Main effect (time) | Main effect (group) |
| Quality of Life | Exercise Control | 69 ± 20 80 ± 17 |  | 74 ± 19 76 ± 24 | +5 -4 |  | F = .58, p = .45, partial η2 = .03 | F = .11, p = .74, partial η2 = .005 | F = .9, p = .35, partial η2 = .04 |
| Physical Functioning | Exercise Control | 99 ± 3 93 ± 13 |  | 97 ± 5 93 ± 12 | -2 0 |  | F = .35, p = .56, partial η2 = .02 | F = .34, p = .56, partial η2 = .02 | F = 1.6, p = .21, partial η2 = .07 |
| Role Functioning | Exercise Control | 92 ± 18 85 ± 26 |  | 95 ± 16 92 ± 15 | +3 +7 |  | F = .28, p = .60, partial η2 = .01 | F = 1.8, p = .19, partial η2 = .08 | F = .48, p = .50, partial η2 = .02 |
| Emotional Functioning | Exercise Control | 79 ± 14 79 ± 22 |  | 86 ± 14 79 ± 19 | +7 0 |  | F =2.3, p = .15, partial η2 = .10 | F =2.3, p = .15, partial η2 = .10 | F = .26, p = .62, partial η2 = .01 |
| Cognitive Functioning | Exercise Control | 90 ± 16 86 ± 22 |  | 92 ± 12 83 ± 19 | +2 -3 |  | F =1.1, p = .30, partial η2 = .05 | F =.06, p = .82, partial η2 = .003 | F =.70, p = .41, partial η2 = .03 |
| Social Functioning | Exercise Control | 92 ± 14 85 ± 26 |  | 95 ± 11 87 ± 23 | +3 +2 |  | F =.05, p = .83, partial η2 = .002 | F =2.8, p = .11, partial η2 = .12 | F =.80, p = .38, partial η2 = .04 |
| Fatigue | Exercise Control | 19 ± 17 21 ± 21 |  | 16 ± 19 23 ± 25 | -3 +2 |  | F =.53, p = .48, partial η2 = .03 | F =.06, p = .82, partial η2 = .003 | F =.38, p = .54, partial η2 = .02 |
| Nausea and Vomiting | Exercise Control | 0 ± 0 1 ± 5 |  | 2 ± 5 1 ± 5 | +2 0 |  | F =.41, p = .53, partial η2 = .02 | F =.41, p = .53, partial η2 = .02 | F =.13, p = .72, partial η2 = .01 |
| Pain | Exercise Control | 3 ± 7 19 ± 19^$^ |  | 7 ± 12 26 ± 29^$^ | +4 +7 |  | F =.12, p = .74, partial η2 = .01 | F =1.2, p = .29, partial η2 = .05 | **F =6.6, p = .02, partial η2 = .24** |
| Dyspnoea | Exercise Control | 10 ± 16 8 ± 15 |  | 7 ± 14 10 ± 21 | -3 +2 |  | F =.43, p = .64, partial η2 = .03 | F =.01, p = .92, partial η2 = .001 | F =.01, p = .92, partial η2 = .001 |
| Insomnia | Exercise Control | 27 ± 26 28 ± 36 |  | 37 ± 29 26 ± 24 | +10 -2 |  | F =1.5, p = .23, partial η2 = .07 | F =.54, p = .47, partial η2 = .03 | F =.18, p = .68, partial η2 = .01 |
| Appetite Loss | Exercise Control | 3 ± 11 3 ± 9 |  | 3 ± 11 8 ± 15 | 0 +5 |  | F =.76, p = .39, partial η2 = .04 | F =.76, p = .39, partial η2 = .04 | F =.22, p = .65, partial η2 = .01 |
| Diarrhoea | Exercise Control | 7 ± 14 8 ± 20 |  | 0 ± 0 18 ± 38 | -7 +10 |  | F =2.5, p = .13, partial η2 = .11 | F =.74, p = .11, partial η2 = .01 | F =1.3, p = .27, partial η2 = .06 |

*Time x group analysis reports the interaction and main effects from two-way repeated measures ANOVA. Data are mean ± SD. Degrees of freedom were F(1,21) in all. ^$^ indicates significant main effect of group, suggesting there is a difference between groups at pre- and post-intervention, but no difference over time at p < 0.05.*

| Supplementary Table 2B. Intervention related changes to the CLL-specific Quality of life (QLQ-CLL16) questionnaire. | | | | | | | | | |
| --- | --- | --- | --- | --- | --- | --- | --- | --- | --- |
|  |  | Time point | | | |  | Time x Group Analysis | | |
|  | Group | Pre-Intervention |  | Post-Intervention | Δ |  | Interaction (time x group) | Main effect (time) | Main effect (group) |
| Disease and Treatment Effects | Exercise Control | 7 ± 10 15 ± 16 |  | 8 ± 8 18 ± 16 | +1 +3 |  | F =.11, p = .75, partial η2 = .01 | F =1.3, p = .26, partial η2 = .06 | F =2.8, p = .11, partial η2 = .12 |
| CLL specific Fatigue | Exercise Control | 18 ± 21 21 ± 25 |  | 8 ± 12 22 ± 28 | -10 +1 |  | F =2.8, p = .11, partial η2 = .12 | F =1.7, p = .21, partial η2 = .07 | F =.74, p = .40, partial η2 = .03 |
| Infection | Exercise Control | 5 ± 3 10 ± 8 |  | 3 ± 4 6 ± 7 | -2 -4 |  | F =.80, p = .38, partial η2 = .04 | F =3.8, p = .06, partial η2 = .16 | F =2.7, p = .11, partial η2 = .12 |
| Social Problems | Exercise Control | 3 ± 11 8 ± 28 |  | 3 ± 11 5 ± 18 | 0 -3 |  | F =.24, p = .63, partial η2 = .01 | F =.24, p = .63, partial η2 = .01 | F =.16, p = .69, partial η2 = .01 |
| Future Health | Exercise Control | 27 ± 14 28 ± 33 |  | 23 ± 22 38 ± 36 | -4 +10 |  | F =3.5, p = .08, partial η2 = .14 | F =.90, p = .35, partial η2 = .04 | F =.53, p = .48, partial η2 = .02 |

*Interaction and main effects from two-way repeated measures ANOVA are reported. Data are mean ± SD. Degrees of freedom were F(1,21) in all.*

| Supplementary Table 2C. Intervention related changes to the FACIT-Fatigue scale, Satisfaction with life scale, sleep quality index (PSQI) and perceived stress scale. | | | | | | | | | |
| --- | --- | --- | --- | --- | --- | --- | --- | --- | --- |
|  |  | Time point | | | |  | Time x Group Analysis | | |
|  | Group | Pre-Intervention |  | Post-Intervention | Δ |  | Interaction (time x group) | Main effect (time) | Main effect (group) |
| FACIT-fatigue scale | Exercise Control | 40.3 ± 10.5 38.7 ± 12.1 |  | 40.6 ± 10.4 39.9 ± 11.7 | +0.3 +1.2 |  | F =.07, p = .79, partial η2 = .004 | F =.20, p = .66, partial η2 = .01 | F =.06, p = .81, partial η2 = .003 |
| Satisfaction with life scale | Exercise Control | 22.6 ± 7 24.5 ± 7.7 |  | 23.6 ± 8 24.1 ± 8.1 | +1 - 0.4 |  | F =.64, p = .44, partial η2 = .03 | F =.09, p = .77, partial η2 = .004 | F =.15, p = .70, partial η2 = .007 |
| Sleep Quality (PSQI) | Exercise Control | 5.3 ± 2.3 5.9 ± 4.7 |  | 5.8 ± 2.9 5.2 ± 3.5 | +0.5 - 0.7 |  | F =1.4, p = .24, partial η2 = .06 | F =.07, p = .80, partial η2 = .003 | F =.0, p = .99, partial η2 = .0 |
| Perceived stress scale | Exercise Control | 12.8 ± 5.3 13.5 ± 7 |  | 12 ± 4.9 13.3 ± 9.7 | -0.8 - 0.2 |  | F =.05, p = .83, partial η2 = .002 | F =.15, p = .71, partial η2 = .007 | F =.14, p = .71, partial η2 = .007 |

*Interaction and main effects from two-way repeated measures ANOVA are reported. Data are mean ± SD. Degrees of freedom were F(1,21) in all except for the FACIT-fatigue scale and the satisfaction with life scale variables which were F(1,20) due to missing data from n = 1 participant that did not complete the FACIT-fatigue scale and the satisfaction with life scale test at post-intervention, therefore the pre-intervention was removed from the analysis. FACIT = Functional assessment of chronic illness therapy questionnaire; PSQI = Pittsburgh sleep quality index.*
