# Supplementary material for: A 16-week progressive exercise training intervention in treatment-naïve chronic lymphocytic leukaemia: a randomised-controlled pilot study
Source: Front Oncol. 2024 Dec 5;14:1472551. doi: 10.3389/fonc.2024.1472551 (PMC11655450; doi:10.3389/fonc.2024.1472551)

Supplemental Figure 1. Representative B-CLL flow cytometry analysis with a sample from a trial participant.

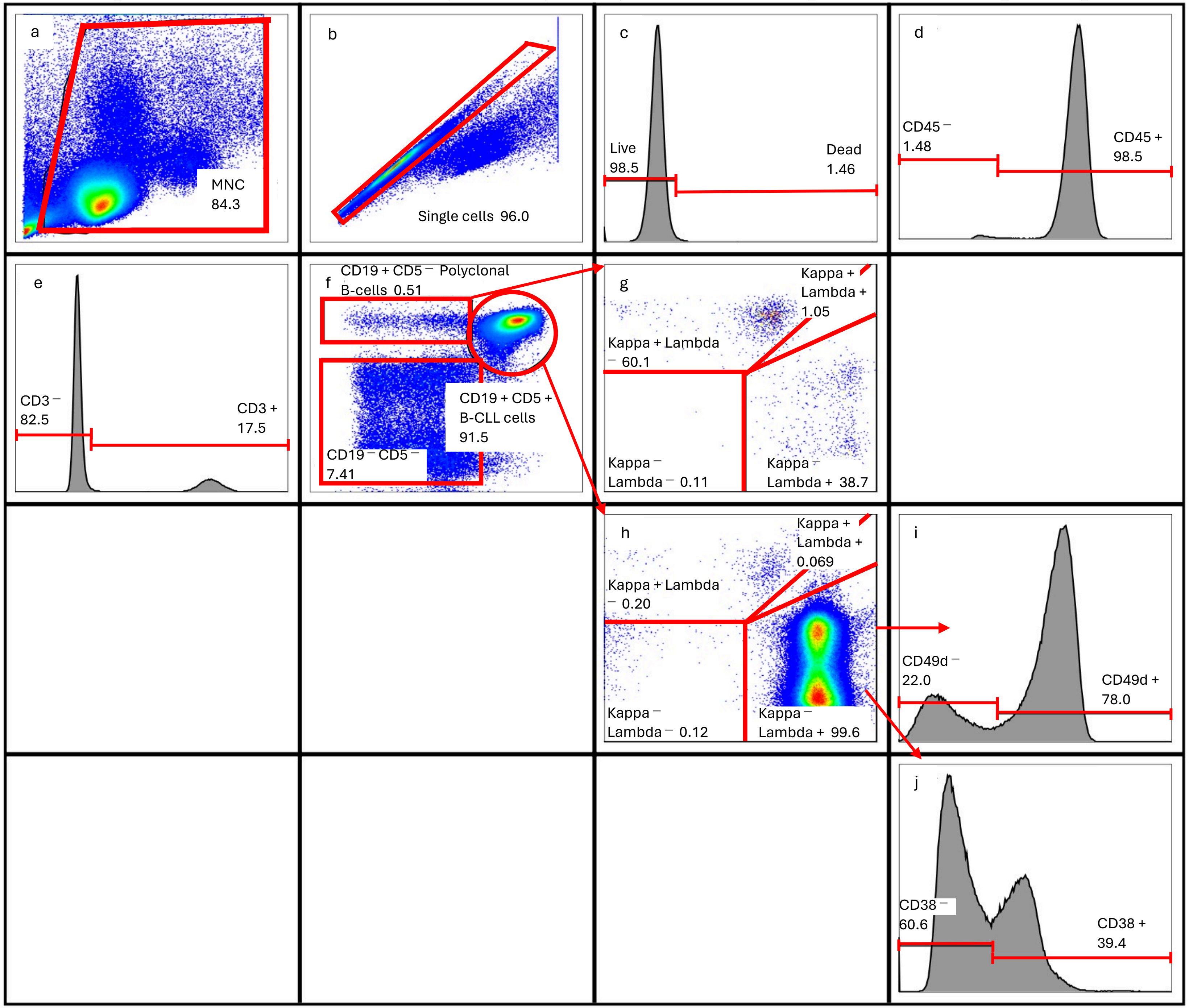

Supplemental Figure 2. Representative CD8+ T-cell flow cytometry analysis with a sample from a trial participant.

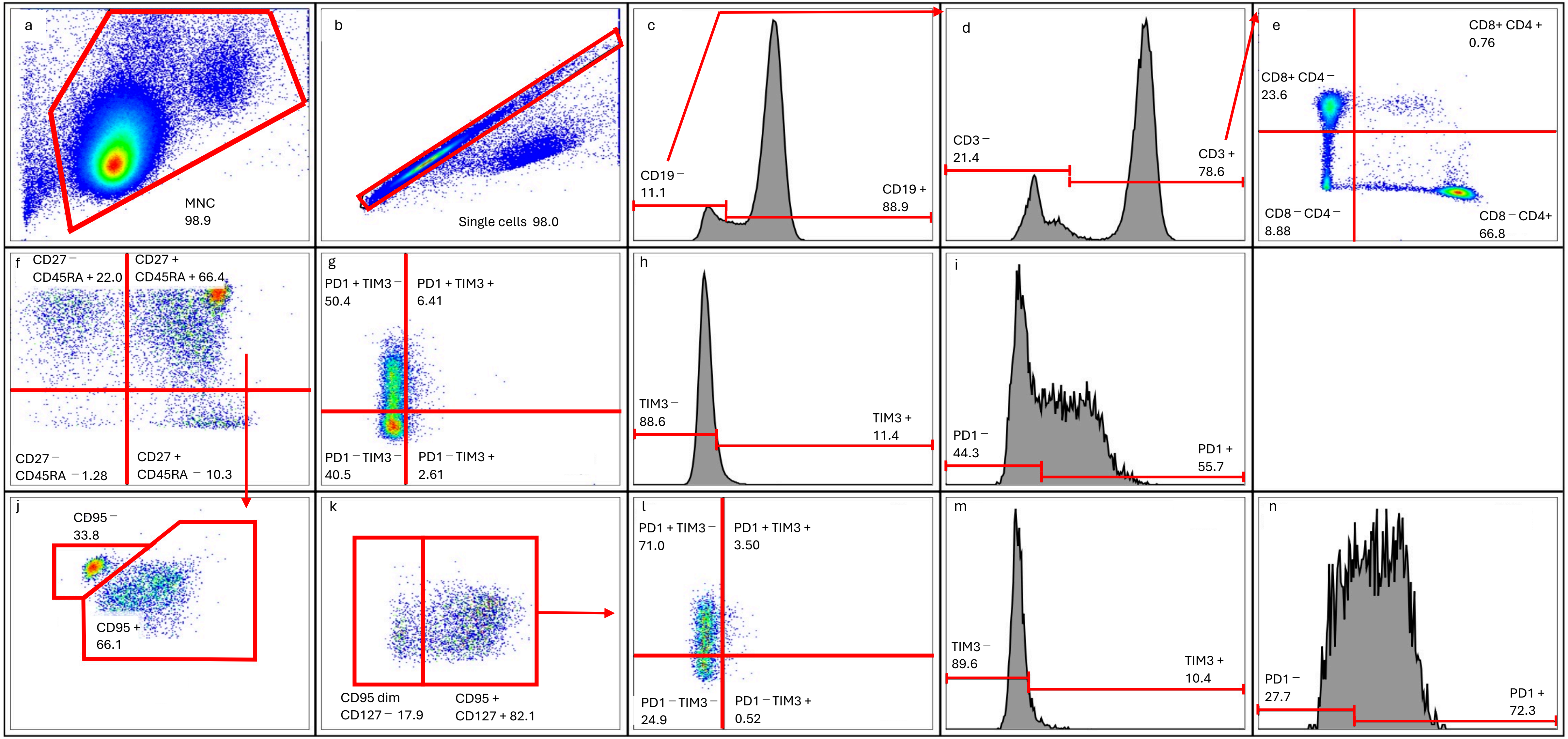

Supplemental Figure 3. Representative CD4+ T-cell flow cytometry analysis with a sample from a trial participant.

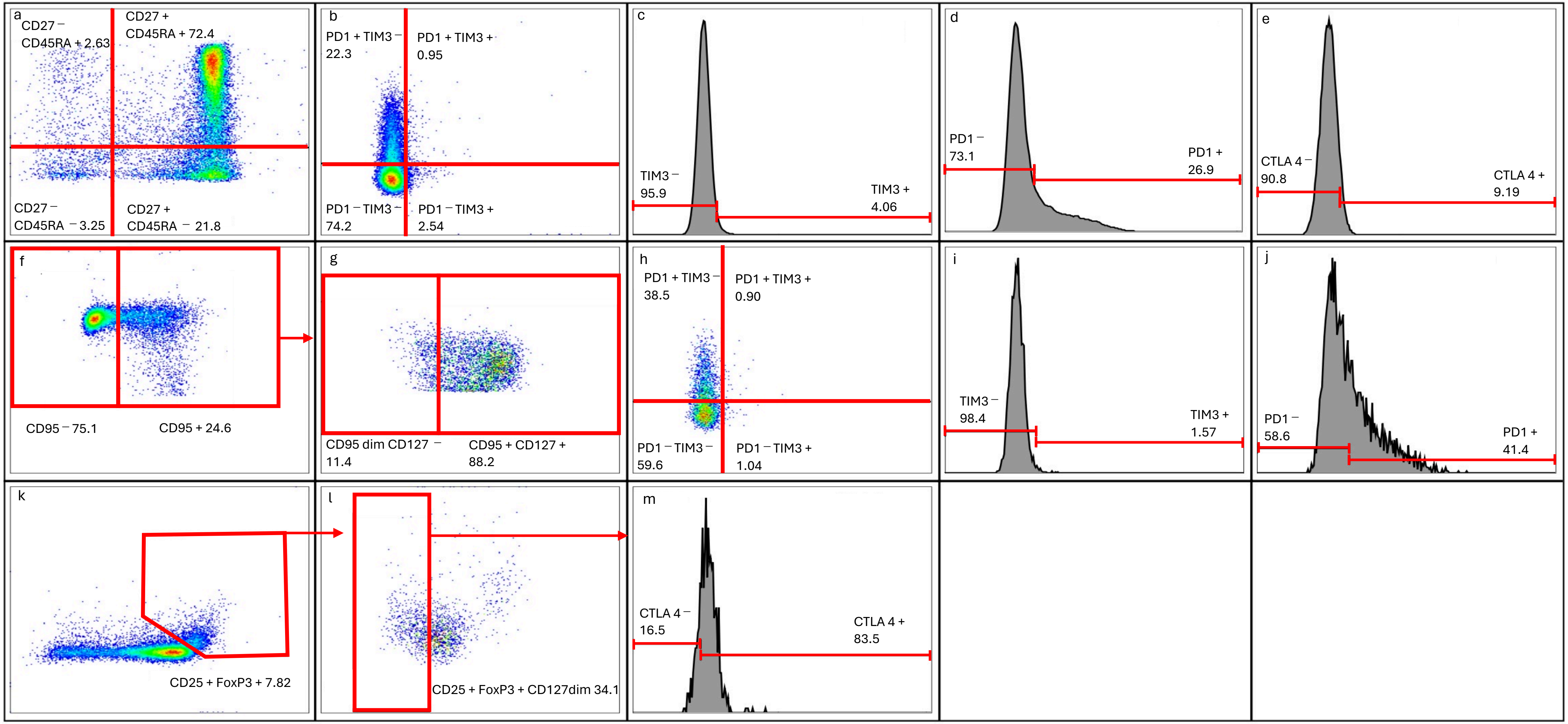

Supplemental Figure 4. Schematic diagram showing the structure of supervised and unsupervised exercise sessions, for the 16-week exercise program.

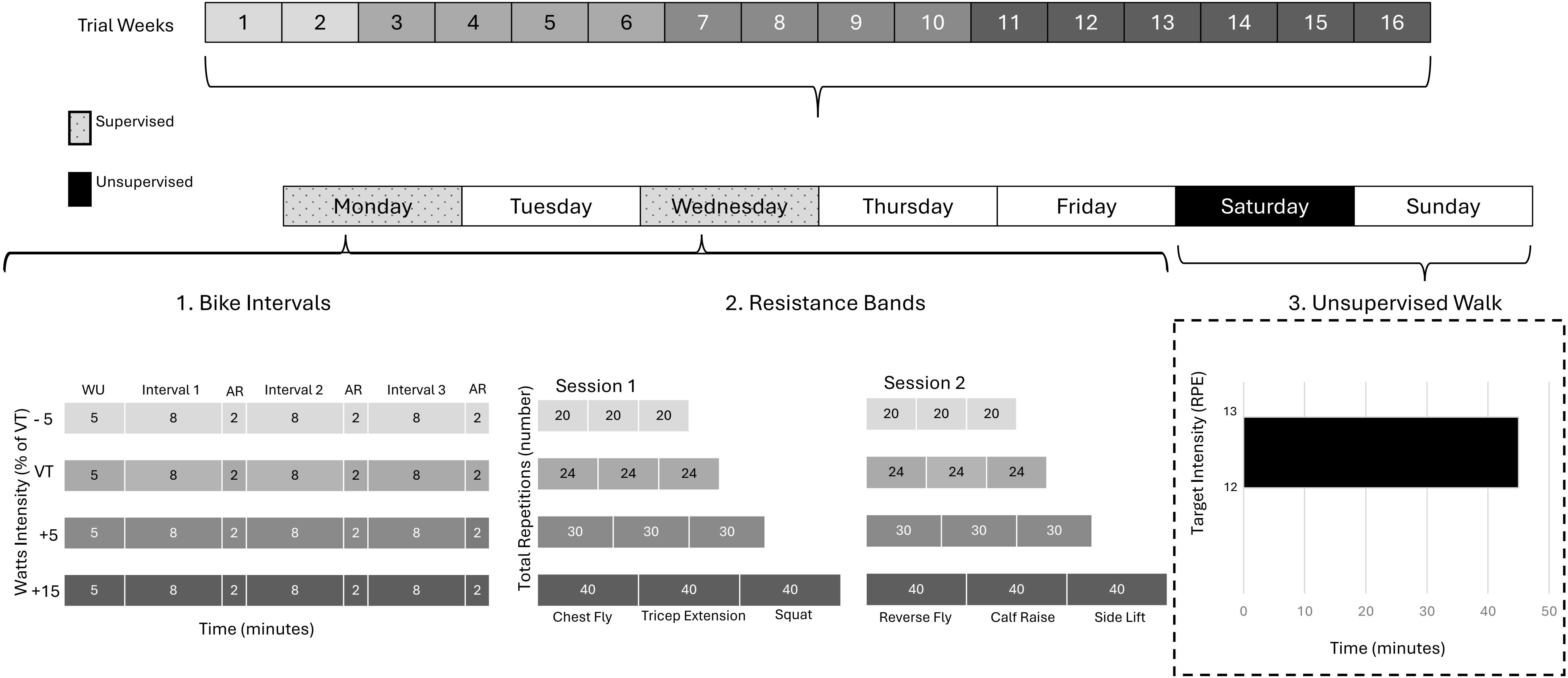

Supplemental Figure 5. Pre- to post-intervention changes to CLL cells.

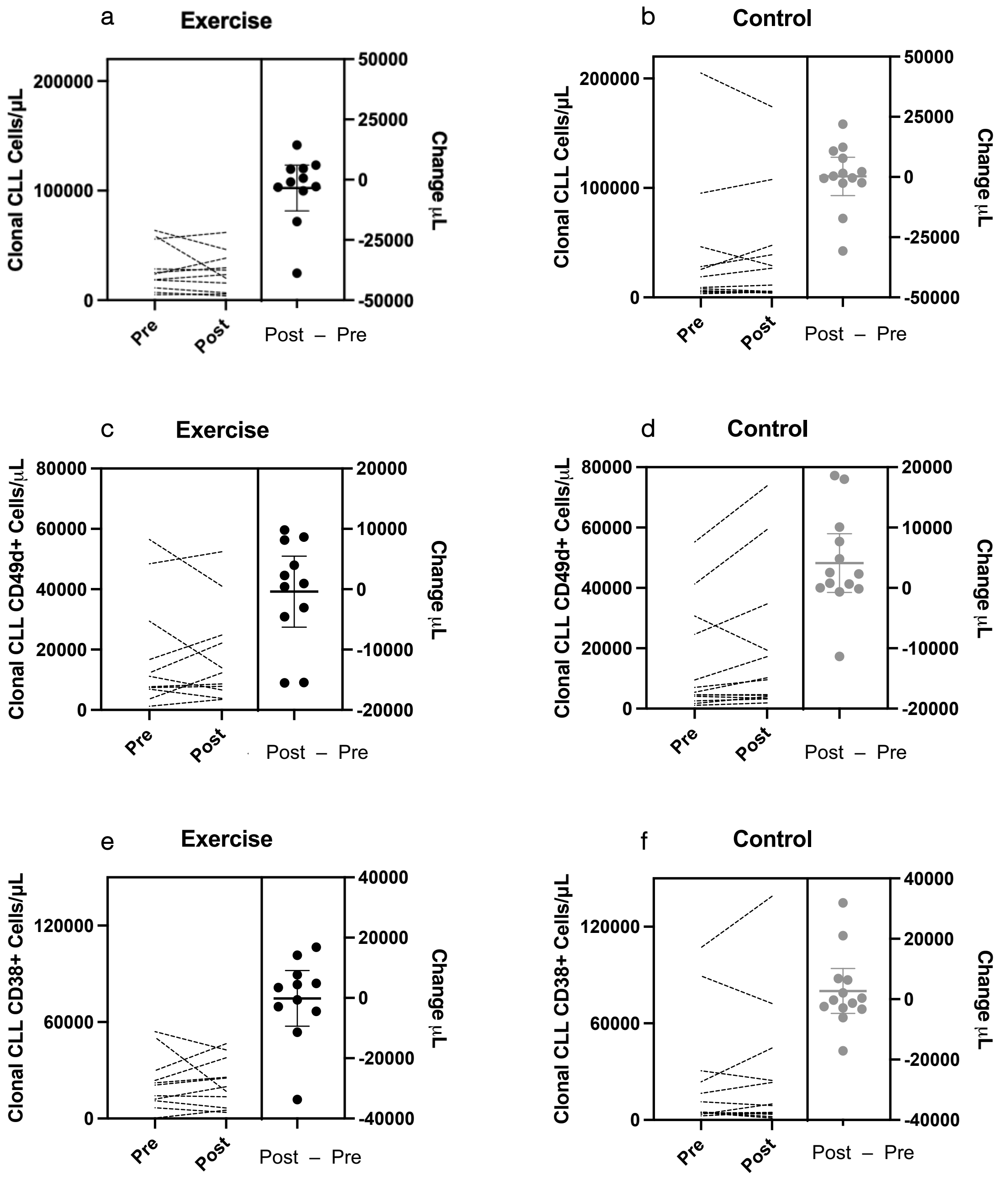

Supplement: Supplementary Figure 1 — Representative B-CLL flow cytometry analysis with a sample from a trial participant. FSC versus SSC was plotted to identify Mononuclear cells (MNC) (A). Singlets were gated to discard doublets (B). Viable cells were identified (C). CD45+ cells were identified (D). CD3+ T-cells were discarded (E). To identify B-CLL cells (CD19+ CD5+) and polyclonal B-cells (CD19+ CD5−) CD19 was gated against CD5 in the CD3− population (F). Within the polyclonal B-cell population kappa was gated against lambda to identify the polyclonal B-cells with restriction to either kappa or lambda (G). The gate drawn in the polyclonal B-cell population was then copied to the B-CLL cell population to identify the clonally restricted B-CLL cells (H). Prognostic markers CD49d+ (I) and CD38+ (J) were also identified in the clonally restricted B-CLL cells. [file Image1.pdf]
